# Maresin1 ameliorates MSU crystal-induced inflammation by upregulating Prdx5 expression

**DOI:** 10.1186/s10020-023-00756-w

**Published:** 2023-11-23

**Authors:** Hui Jiang, DianZe Song, Xiaoqin Zhou, Feng Chen, Qingqing Yu, Long Ren, Qian Dai, Mei Zeng

**Affiliations:** 1 Institute of Rheumatology and Immunology, The Affiliated Hospital of North Sichuan Medical College, 1# South Maoyuan Road, Nanchong, 637001 Sichuan Province China; 2 Institute of Basic Medicine and Forensic Medicine, North Sichuan Medical College, 234# Fujiang Road, Nanchong, 637001 Sichuan Province China; 3 Medical Imaging Key Laboratory of Sichuan Province, The Affiliated Hospital of North Sichuan Medical College, 1# South Maoyuan Road, Nanchong, 637001 Sichuan China; 4 The Fifth People’s Hospital of Nanchong City, 21# Bajiao Street, Nanchong, 637100 Sichuan China

**Keywords:** MSUc, MaR1, Prdx5, Keap1-Nrf2 signaling axis, FAO

## Abstract

**Background:**

Maresin1 (MaR1) is a potent lipid mediator that exhibits significant anti-inflammatory activity in the context of several inflammatory diseases. A previous study reported that MaR1 could suppress MSU crystal-induced peritonitis in mice. To date, the molecular mechanism by which MaR1 inhibits MSU crystal-induced inflammation remains poorly understood.

**Methods:**

Mousebone marrow-derived macrophages (BMDMs) were pretreated with MaR1 and then stimulated with FAs (palmitic, C16:0 and stearic, C18:0) plus MSU crystals (FAs + MSUc). In vivo, the effects of MaR1 treatment or Prdx5 deficiency on MSUc induced peritonitis and arthritis mouse models were evaluated.

**Results:**

The current study indicated that MaR1 effectively suppressed MSUc induced inflammation in vitro and in vivo. MaR1 reversed the decrease in Prdx5 mRNA and protein levels induced by FAs + MSUc. Further assays demonstrated that MaR1 acceleratedPrdx5 expression by regulating the Keap1-Nrf2 signaling axis. Activation of AMPK by Prdx5 improved homeostasis of the TXNIP and TRX proteins and alleviated mitochondrial fragmentation. In addition, Prdx5 overexpression inhibited the expression of CPT1A, a key enzyme for fatty acid oxidation (FAO). Prdx5 protected against defects in FA + MSUc induced FAO and the urea cycle.

**Conclusion:**

MaR1 treatment effectively attenuated MSUc induced inflammation by upregulating Prdx5 expression. Our study provides a new strategy by which Prdx5 may help prevent acute gout attacks.

**Supplementary Information:**

The online version contains supplementary material available at 10.1186/s10020-023-00756-w.

## Introduction

Gout is the most common type of inflammatory arthritis in men. Global epidemiology data show that the incidence and prevalence of gout are increasing in both developed and developing countries (Kuo et al. 2015). Hyperuricemia leads to the deposition of monosodium urate crystals (MSUc), which induces recurrent episodes of acute joint inflammation (Faires and McCarty 1962). MSUc are damage-associated molecules that can activate innate immune pathways. However, MSUc are present in some asymptomatic hyperuricemic individuals and do not trigger an acute inflammatory response, suggesting that other factors may act synergistically with MSUc to induce inflammation (Dalbeth et al. 2016). The activation of the NOD-like receptor thermal protein domain associated protein 3 (NLRP3) inflammasome by MSUc in macrophages and monocytes is closely related to the initiation of gout flares (Martinon et al. 2006). After the involvement of the NLRP3 inflammasome in gout attacks was demonstrated, it was thought that only MSUc were responsible for the formation and activation of the NLRP3-ASC-caspase 1 protein complex, which performs proteolysis of pro-IL-1β to active IL-1β (Martinon et al. 2002). Damage-associated molecular patterns (such as free fatty acids) and microbial products (such as lipopolysaccharide) have been identified as signals that upregulate IL-1β mRNA and pro-IL-1β protein levels (Giamarellos-Bourboulis et al. 2009; Joosten et al. 2010). During the initiation phase of a gout flare, the NLRP3 inflammasome mediates the production of mature IL-1β and appears to be important (Dalbeth et al. 2021).

Several studies have reported that a variety of chemical mediators derived from ω-3 polyunsaturated fatty acids (ω-3 PUFAs) can alleviate the acute inflammatory response (Jin et al. 2018). These mediators include so-called specialized proresolving lipid mediators such as lipoxins, resolvins, protectins and maresins (MaRs) (Serhan 2014). MaR1 is produced from docosahexaenoic acid (DHA) in macrophages (Buckley et al. 2014). MaR1 treatment greatly decreased cytokine IL-1β secretion in several LPS-induced disease models (Gu et al. 2018; Zhang et al. 2017). Recent studies have shown that during acute inflammation, MaR1 suppresses neutrophil infiltration, accelerates macrophage efferocytosis, and promotes tissue regeneration (Dalli et al 2013). It has been demonstrated that MaR1 represses NLRP3 inflammasome activation by upregulating NLRP3 ubiquitination and further attenuates caspase-1 activation and IL-1β secretion (Zheng et al. 2020). Several studies have demonstrated that MaR1 has the potential to inhibit the NF-κB pathway. NF-κB activation promotes IL-1β and NLRP3 expression at the transcriptional level (Zhang et al. 2017; Qiu et al. 2020). All these findings prompted us to investigate the effect of MaR1 on MSU crystal-induced inflammation and its underlying molecular mechanism.

In this study, we first examined the effects of MaR1 on the MSU crystal-induced inflammatory response and mitochondrial function in vitro, followed by an exploration of the molecular mechanisms of MaR1 anti-inflammation. MaR1 accelerated Prdx5 expression, as confirmed by proteomics and Western blotting assay. As an important member of the Peroxiredoxin (Prdx) family, Prdx5 has antioxidant protection function and can effectively inhibit reactive oxygen species (ROS) generation. Therefore, we further investigated the role of Prdx5 on mitochondrial dynamic balance and metabolic defect induced by MSUc. We also have demonstrated in mouse models that MaR1 can effectively inhibit MSUc-induced inflammatory responses and that Prdx5 plays a crucial role in this process.

## Materials and methods

### Mice

C57BL/6 and Prdx5 knockout (C57BL/6JSmoc-Prdx5 em1Smoc) mice were obtained from Shanghai Model Organisms (China). All animal procedures were approved by the North Sichuan Medical College Animal Care and Use Committee.

### MSU crystal formation

MSUc were prepared as previously described (Dalbeth et al. 2008) and suspended at a concentration of 25 mg/mL in sterile, endotoxin-free phosphate-buffered saline (PBS).

### Preparation of palmitic acid and stearic acid

Palmitic acid and stearic acid (fatty acids, FAs) were synthesized as previously described (Hall et al. 2018). The details preparation of palmitic and stearic acids are provided in the supplementary materials.

### Bone marrow-derived macrophage culture, MaR1 treatment and FAs + MSUc stimulation

Primary bone marrow-derived macrophages (BMDMs) were obtained by culturing bone marrow cells from C57BL/6 or Prdx5^−/−^ mice at 8–10 weeks of age (either sex). The BMDMs were cultured in Dulbecco’s modified Eagle’s medium (DMEM) supplemented with 10% fetal bovine serum (FBS) and 100 U/mL penicillin‒streptomycin in the presence of macrophage colony-stimulating factor (M-CSF) (10 ng/mL) (R&D Systems) for 5–7 days. BMDMs were treated with MaR1 (10 nM) for 1 h and then stimulated with FAs (50 μM) + MSUc (50 μg/mL) for 12 h.

### DIA proteomics and bio-informatics analysis

Data independent acquisition (DIA) proteomics analysis was performed on BMDMs treated with or without MaR1 for 1 h and then stimulated with FAs + MSUc (each group n = 3). This process was carried out at Shanghai Applied Protein Technology Co., Ltd. The details of DIA proteomics and Bio-informatics analysis are listed in Additional file 1.

### Western blot analysis

Western blot assay was performed according to standard protocols. Cells were lysed using RIPA buffer, and then protein concentration was detected by bicinchoninic acid (BCA) protein assay kits (Beyotime). After separated by SDS-PAGE electrophoresis, proteins were transferred onto a polyvinylidene fluoride (PVDF) membrane (BioRad), and blocked with 5% bovine serum albumin (BSA) solution. The membranes were incubated with primary antibodies in 4 ℃ overnight and secondary antibodies in room temperature for 1 h. Finally, the membranes were imaged. The information about the antibodies used in the Western blot assay was shown in Additional file 1: Table S1.

### Prdx5 overexpression or Keap1 knockdown in BMDMs

BMDMs were plated in 6-well plates (5 × 10^5^ cells per well) and then transfected with 1.5 μg plasmid or 100 pM siRNA using a Lipo8000™ kit (Beyotime, China) according to the manufacturer’s guidelines. The plasmid pcDNA3.1 (Ctrl) or Prdx5 open reading frame (ORF) inserted in the pcDNA3.1 plasmid (Prdx5) was ordered from Nanjing Jingmai Biotech Co. Ltd. (China). The Keap1 siRNA sequences are listed in Additional file 1: Table S2.

### RNA isolation, cDNA synthesis and quantitative PCR (qPCR)

BMDMs were dissolved in TRIzol reagent (TIANGEN, China). cDNA was synthesized from total RNA using a reverse transcriptase kit (Vazyme, China) following the manufacturer’s protocol. Quantitative PCR (qPCR) was performed with SYBR Green premix (Vazyme, China) and detected with a real-time PCR system (Applied Biosystems). GAPDH was used as an internal control gene. The sequences of all primers are shown in Additional file 1: Table S3.

### ELISA

Supernatants from cell cultures or peritoneal lavage fluid were evaluated for mouse IL-1β, IL-6 and TNF-α (BOSTER, China) following the manufacturer’s instructions.

### Immunofluorescence

BMDMs were cultured on cover slips overnight, and then the corresponding interventions were performed. After washing three times with PBST (1% Tween 20 in PBS solution), the cells were fixed with 4% poly formaldehyde (PFA) in PBS for 10 min and then washed five times with PBST. After permeabilization with Triton X-100 (0.3% Triton X-100 in PBS solution) and blocking with 10% goat serum/PBS, the cells were incubated with the corresponding primary antibody overnight at 4 °C. After washing with PBST, the cells were incubated with secondary antibodies in blocking solution for 60 min and rinsed in PBST. LCM imaging was performed using an Olympus FV3000. At least 60 cells were analyzed for each slide. The information about the antibodies used in the immunofluorescence assay was supplied in Additional file 1: Table S1.

### Metabolite profiling analysis

BMDMs were cultured as indicated and collected. Then, metabolites were extracted and quantified by LC/MS analysis at Wuhan MetWareBiotech Co. Ltd. (China). The details of metabolites profiling analysis are supplied in Additional file 1.

### Transmission electron microscopy (TEM)

After the indicated treatment, 2 × 10^6^ cells were fixed in 4% glutaraldehyde (Santa Cruz, CA, USA) for 24 h at 4 °C. Samples were sent to Chengdu LiLai Biotech Co. Ltd. (China) for subsequent processing and image acquisition.

### Mitochondrial membrane potential (MMP, *Ψ*m), mitochondrial ROS (mtROS), mitochondrial permeability transition pore (mPTP)and mitochondrial Calcium detection

*Ψ*m was evaluated using a JC-1 probe (Beyotime, C2003S). BMDMs were incubated with JC-1 probe for 20 min at 37 °C. The cells were then evaluated by flow cytometry (FCM) and the red/green ratio was used to show Δψm alterations. MitoSOX^TM^Red (Molecular Probes, M36008) is often used to evaluate mitochondrial ROS. Briefly, BMDMs were incubated with MitoSOX™ Red (1.25 μM) at 37 °C for 30 min. After washing three times with PBS, BMDMs were evaluated using FCM, and their MitoSOX (%) was analyzed. Calcein AM (Beyotime, C2009S) was used to detect mPTP. BMDMs were stained with Calcein (0.5 ×) and CoCl_2_ (1 ×), and then detected respectively through FCM and LCM. Mitochondrial Ca^2+^ concentration was examined by Rhod-2/AM. BMDMs were incubated with Rhod-2/AM (2.5 μM) and PluronicF127 (0.05%) for 30 min, then cell nucleus were stained with DAPI (0.5 μg/ml). The fluorescence signal intensity was analyzed by LCM.

### Mitochondrial morphology evaluation

For live cell imaging, mitochondrial morphology was analyzed by staining with 100 nM MitoTracker Green probe (Beyotime, China, C1048) and imaging by Laser Confocal Microscope (LCM, Olympus FV3000). The aspect ratio (AR) was determined as the ratio between the major axis and minor axis of each mitochondrion. Mitochondria from at least 100 cells were analyzed.

### Molecular modeling study of MaR1 with Keap1

The molecular modelling study of MaR1 with Keap1 was carried out using AUTODOCK 4.2. The details of molecular modeling study were supplied in Additional file 1.

### MSU crystal-induced joint inflammation and peritonitis

MSU crystal-induced arthritis and peritonitis mouse models were generated as previously described (Lv et al. 2021).The details of MSU crystal-induced joint inflammation and peritonitis were shown in the supplementary materials. MPO activity, as a quantitative assay for neutrophil aggregation, was measured in footpad tissue homogenates using the MPO Colorimetric Assay Kit (Jiancheng Technology Co., Ltd., Nanjing, China) according to the manufacturer's instructions.

### Statistical analysis

All values are presented as the mean ± standard deviation (SD). Statistical analysis was conducted using one-way ANOVA. All statistical analyses were evaluated using GraphPad Prism software (Version 6.0). Differences were considered significant if the P value was less than 0.05.

## Results

### MaR1 has a strong effect on the expression of inflammatory factors induced by FAs + MSUc

**Fig. 1:**
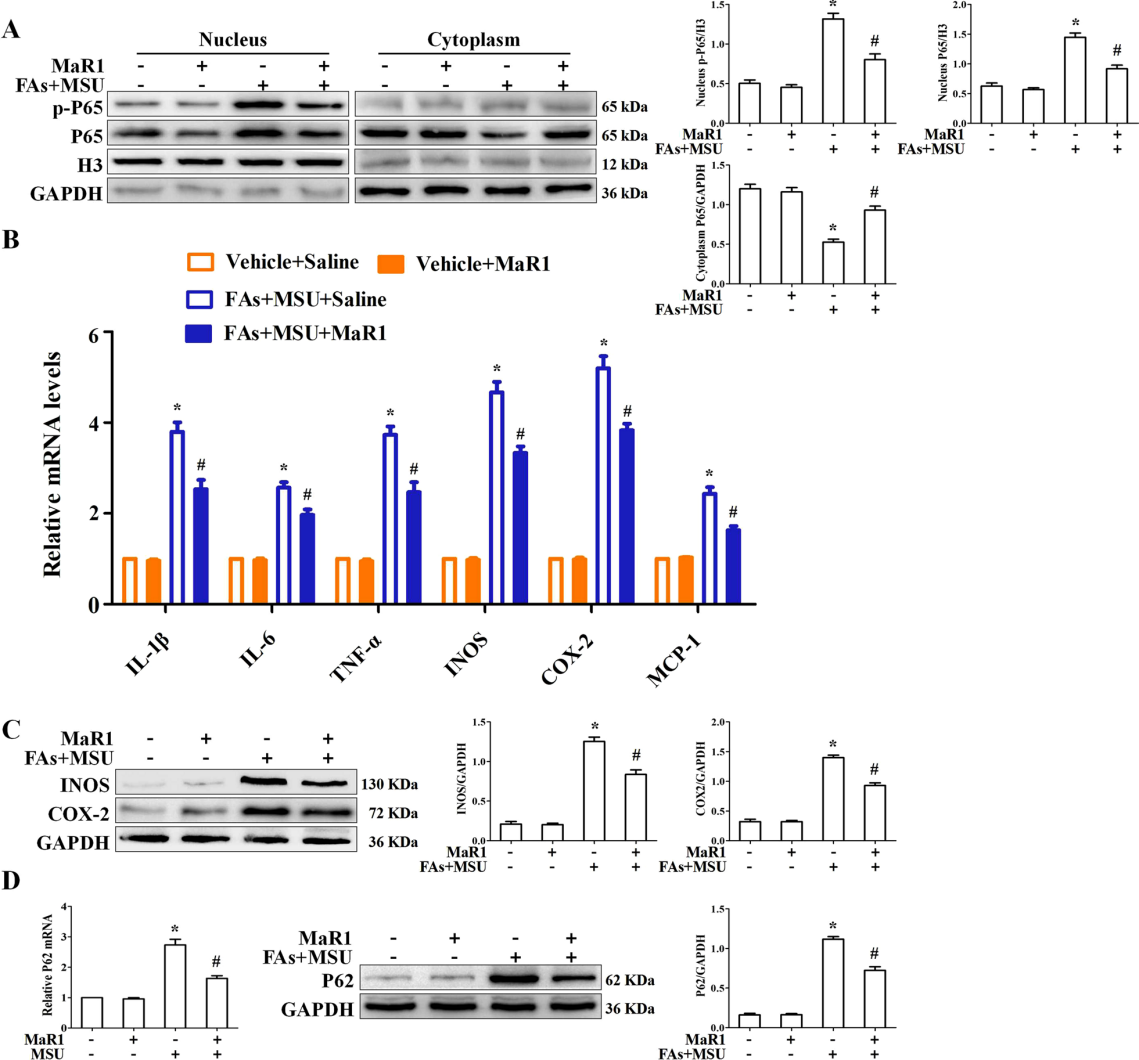
MaR1 inhibited nuclear localization of p-P65 and expression of inflammation-associated genes. **A** BMDMs were treated with or without MaR1 (10 nM) for 1 h, then stimulated with FAs + MSUc for 12 h. Western blot detection of p-P65 and P65 protein levels in the nucleus and cytoplasm. **B** Quantitative PCR for mRNA levels of IL-1β, IL-6, TNF-α, iNOS, COX-2 and MCP1. **C** Western blotting detection of INOS and COX-2 protein levels. **D** Quantitative PCR and Western blot to detect P62 protein level. *Compared with no FAs + MSUc treatment, # compared with FAs + MSUc treatment. * and # means P < 0.05

To understand the anti-inflammatory activity of MaR1, we investigated its effect on NF-κB activation and inflammatory gene expression in BMDMs. BMDMs were incubated with or without MaR1 (10 nM) for 1 h and then stimulated with FAs + MSUc for 12 h. MaR1 significantly inhibited FAs + MSU crystal-induced P65 nuclear translocation (phosphorylation P65, p-P65) (Fig. 1A). Simultaneously, MaR1 decreased the mRNA levels of cytokines, chemokines and pro-inflammatory enzymes, many of which are responsive to NF-κB (Fig. 1B). Consistent with the RT‒qPCR data, MaR1 had similar effects on the secretion of proinflammatory cytokines (Additional file 1: Fig. S1). Moreover, MaR1 attenuated the INOS and COX2 protein levels induced by FAs + MSUc (Fig. 1C). MaR1 also decreased the mRNA and protein expression of the FA + MSU crystal-stimulated autophagic chaperone p62, which is encoded by the NF-κB-inducible Sqstm1 gene (Fig. 1D). These data indicate that MaR1 plays an important role in suppressing MSU crystal-induced inflammation in vitro.

**Fig. 2:**
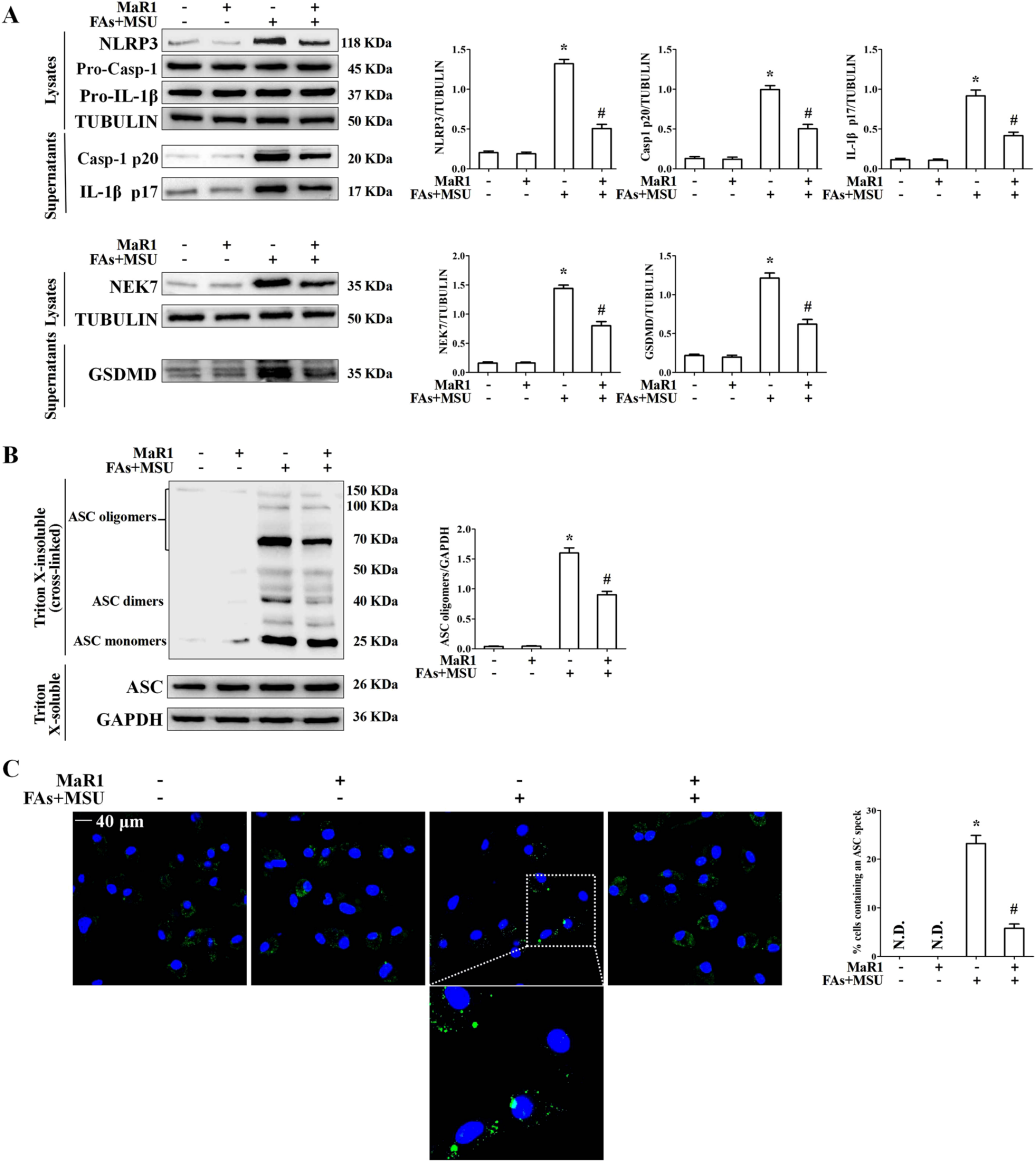
MaR1 inhibited NLRP3 inflammasome activation induced by FAs + MSUc. **A** Western blot for NLRP3, Pro-Casp-1, Pro-IL-1β, IL-1β (p17), Casp1(p20), NEK7 and GSDMD protein levels. **B** Western blotting was used to detect ASC oligomers formation. **C** Immunofluorescence detection of ASC speck formation. DAPI stains nucleus. Scale bar: 40 μm. *Compared with no FAs + MSUc treatment, # compared with FAs + MSUc treatment. * and # means P < 0.05

NLRP3 inflammasome activation plays an essential role in the acute inflammatory response in the context of gout. We demonstrated the effects of MaR1 on pro-IL-1β processing and NLRP3 inflammasome activation. MaR1greatly decreased NLRP3 and NEK7 protein levels but had no effect on pro-IL-1β, ASC and pro-Casp1 protein levels (Fig. 2A). Importantly, MaR1 inhibited Casp1 activation and pro-IL-1β processing to mature IL-1β in BMDMs treated with FAs + MSUc. MaR1 also suppressed gasdermin D (GSDMD) cleavage (Fig. 2A). To validate NLRP3 inflammasome inhibition, we also evaluated MaR1’s effect on ASC oligomerization, an indicator of NLRP3 inflammasome assembly (Dick et al. 2016) (Fig. 2B). Consistent with its effect on Casp1, MaR1 decreased FA + MSU crystal-induced ASC speck formation (Fig. 2C).

### MaR1 protected against mitochondrial dysfunction and mtDNA release induced by FAs and MSUc

**Fig. 3:**
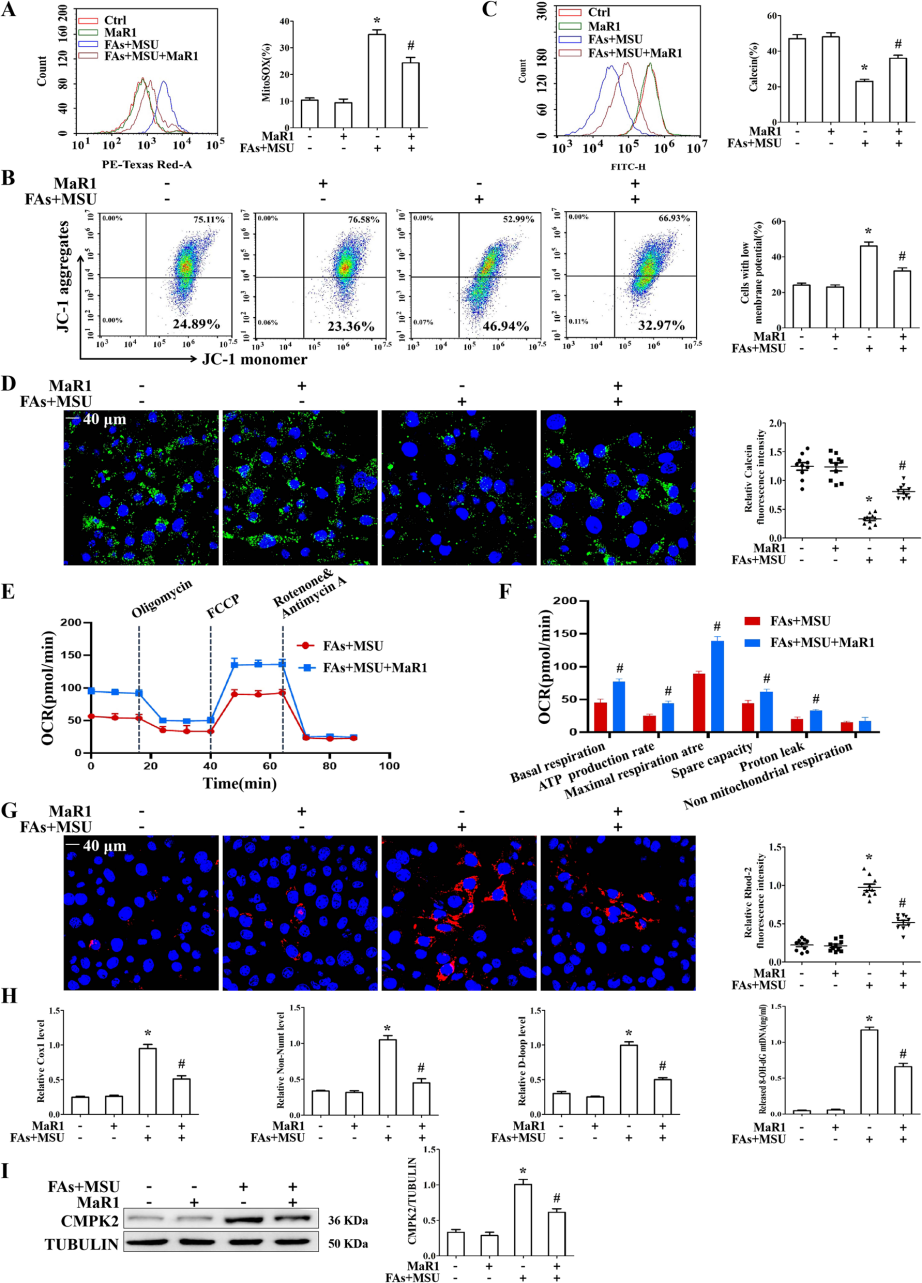
MaR1 protected against MSU crystal-induced mitochondrial dysfunction. **A–C** FCM was used to detect mitochondrial ROS (MitoSOX probe) generation, mitochondrial membrane potential (JC-1 probe) and mPTP (Calcein probe). **D** LCM imaging of mPTP. **E** OCR was measured by Seahorse analysis. **F** Quantification of OCR of basal respiration, ATP production rate, maximal respiration rate, spare capacity, proton leak and non mitochondrial respiration. **G** mitochondrial Ca^2+^ levels. Scale bar: 40 μm. DAPI stains nucleus. **H** Detection of cytoplasmic mitochondrial DNA (mtDNA) and oxidized mtDNA by qPCR and ELISA, respectively. **I** Western blotting was used to detect CMPK2 protein levels. *Compared with no FAs + MSUc treatment, # compared with FAs + MSUc treatment. * and # means P < 0.05

Most NLRP3 inflammasome activators lead to mitochondrial dysfunction, mainly by stimulating mtROS production, inducing mitochondrial membrane depolarization and promoting mitochondrial permeability transition pore (mPTP) opening. FAs + MSUc stimulation induced a significant increase in mitochondrial ROS production, with increased MitoSOX fluorescence intensity (Fig. 3A). JC-1 staining revealed an important shift in fluorescence emission from JC-1 aggregates to JC-1 monomer in response to FAs + MSUc (Fig. 3B). According to the statistical analysis, the JC-1 monomer/JC-1 aggregate ratio associated with the MMP increased from 24% in BMDMs treated with vehicle to 46% in BMDMs treated with FAs + MSUc (Fig. 3B). FAs + MSUc challenge induced mPTP opening, as reflected by a significant decrease in Calcein fluorescence intensity (Fig. 3C). However, in the MaR1 intervention group, the mitochondrial functions of BMDMs were protected, as the level of mitochondrial ROS decreased, and the mPTP closure degree and mitochondrial membrane potential were improved (Fig. 3A–C). By performing live cell imaging, we observed the intracellular Calcein fluorescence intensity, and MaR1 ameliorated the decrease in fluorescence intensity caused by FAs + MSUc stimulation, further indicating that MaR1 inhibited the opening of the mPTP induced by FAs + MSUc (Fig. 3D). To further clarify the effects of MaR1 on mitochondrial function, we utilized Seahorse analysis to test the impact of MaR1 on mitochondrial respiration. As shown in Fig. 3E and F, MaR1 accelerated basal respiration, ATP production rate, maximal respiration rate, spare capacity and proton leak in BMDMs treated with FAs + MSUc.

NLRP3 inflammasome agonists drive mPTP opening caused by an elevated mitochondrial Calcium ion concentration ([Ca^2+^] m), which then drives mtDNA and Ox-mtDNArelease (Xian et al. 2022). We examined the effect of MaR1 on the [Ca^2+^] m. The fluorescence intensity of Rhod-2 (a dye that fluoresces in response to mitochondrial Ca^2+^ binding) in BMDMs stimulated with FAs + MSUc was strongly elevated (Fig. 3G). MaR1 treatment attenuated the increase in the [Ca^2+^] m (Fig. 3G). MaR1 strongly inhibited FAs + MSU crystal-stimulated mtDNA synthesis (Additional file 1: Fig. S2) and blocked the production of cytoplasmic mtDNA and Ox-mtDNA in BMDMs challenged with FAs + MSUc (Fig. 3H). Because CMPK2 controls mitochondrial DNA synthesis (Zhong et al. 2018), we further confirmed the effect of MaR1 on CMPK2 protein levels. MaR1 markedly blocked the upregulation of CMPK2 protein expression induced by FAs + MSUc (Fig. 3I).

### MaR1 accelerated Prdx5 expression in BMDMs treated with FAs + MSUc

**Fig. 4:**
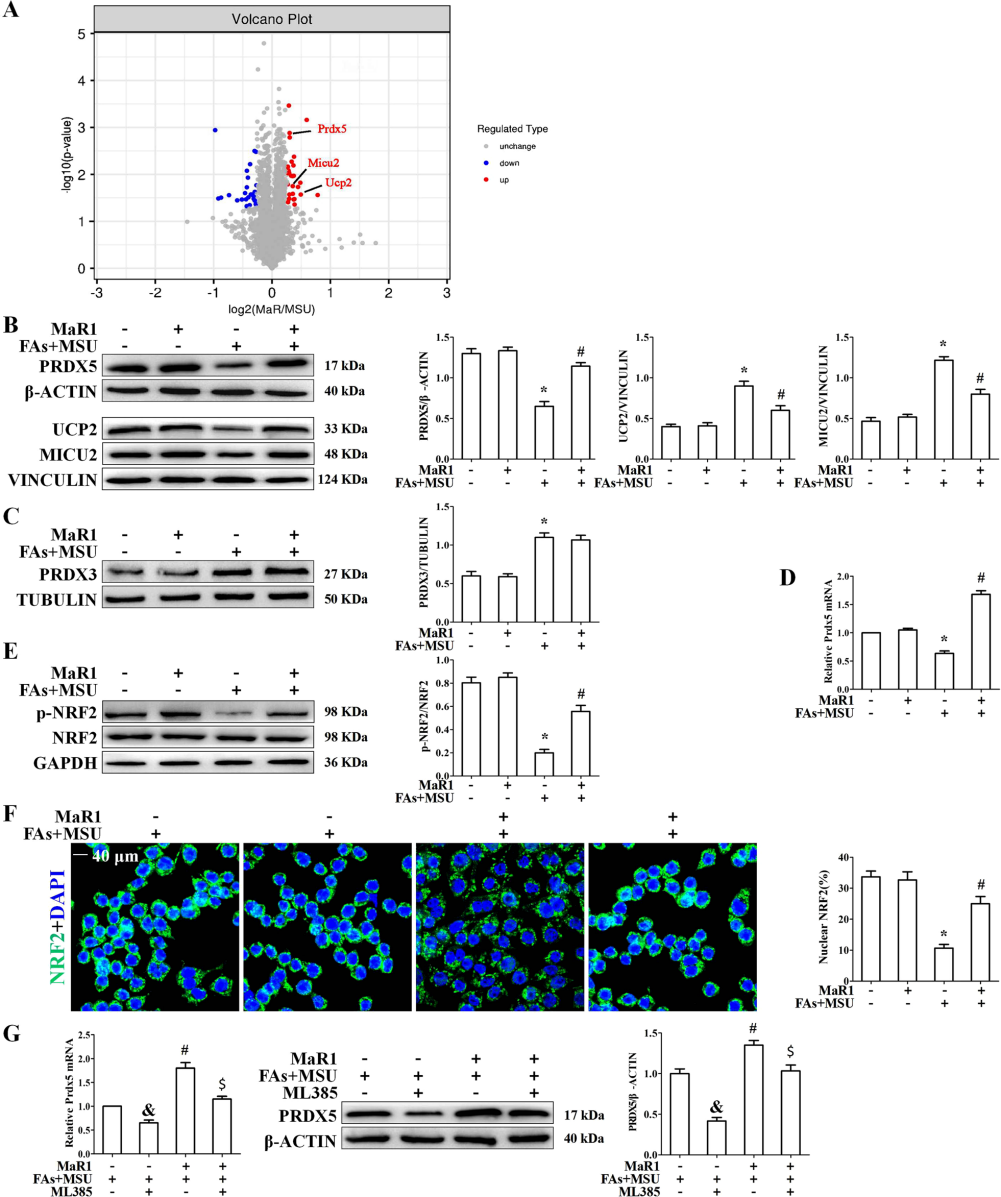
MaR1 promotes Prdx5 expression by accelerating NRF2 activity. **A** DEPs between MaR1-treated and untreated FAs + MSU crystal-stimulated BMDMs are displayed by a volcano plot. The up-regulated proteins in the MaR1 treatment group are shown in red, down-regulated proteins are shown in blue, and those with no difference are shown in gray. **B** Western blot detection of Prdx5, UCP2 and MICU2 protein levels. **C** Western blot detection of Prdx3 protein level. **D** Detection of Prdx5 mRNA level by qPCR. **E** Western blot detection of p-NRF2 protein level. **F** Detection of nuclear localization of NRF2 by immunofluorescence staining. Scale bar: 40 μm. **G** BMDMs were treated with or without ML385 (5 μM) for 1 h, then treated with MaR1 and FAs + MSUc for 12 h. Quantitative PCR and Western blot to detect the effect of ML385 reversing MaR1 on the mRNA and protein levels of Prdx5. & Compared with no MaR1 and ML385 treatment, # compared with FAs + MSUc treatment. $ compared with MaR1 and FAs + MSUc treatment. &, # and $ means P < 0.05

To investigate the molecular mechanisms of MaR1 in MSU crystal-induced inflammatory responses, we performed proteomic and Bioinformatics analyses of differentially expressed proteins (DEPs) in BMDMs treated with or without MaR1 and stimulated with FAs + MSUc. Hierarchal clustering partitioned the differentially expressed proteins (DEPs) into 2 distinct clusters separating BMDMs treated with or without MaR1 and stimulated with FAs + MSUc, implying that the proteomics data were highly reliable (Additional file 1: Fig. S3). A total of 53 differentially expressed proteins (DEPs) between the MaR1-treated and untreated groups were selectedin BMDMs treated with FAs + MSUc by significant difference protein screening, and the information about the genes encoded by the DEPs is provided in Additional file 1: Table S4. Among them, 28 DEPs were upregulated in the MaR1-treated group compared to the MaR1-untreated group, while 25 DEPs were downregulated. The results are illustrated by a volcano plot (Fig. 4A). Three of the 28 upregulated proteins were localized to mitochondria, including Prdx5, UCP2 and MICU2. Western blot assays were used to verify the effect of MaR1 on the expression of these three proteins. The data indicated that MaR1 could effectively alleviate the reduction in Prdx5, UCP2 and MICU2 protein expression induced by FAs + MSUc (Fig. 4B).

Among these upregulated genes, we were particularly interested in Prdx5. Prdx5 belongs to a family of cysteine-dependent peroxidase enzymes with a remarkable ability to scavenge peroxides and peroxynitrite in mammalian cells. There are 6 members of the Prdx family (Prdx1-6), which are classified according to their subcellular localization (Wood et al. 2003). Compared to the other Prdxs, Prdx3 and Prdx5 are more strongly mitochondrial-targeted antioxidant enzymes because they are mainly localized in the mitochondria (Poynton and Hampton 2014). Prdx3 and Prdx5 can effectively scavenge cytosolic and mitochondrial ROS in several cell lines (Huh et al. 2012; Park et al. 2016). We confirmed the effects of FAs + MSUc on the protein levels of Prdx1-6. As shown in Additional file 1: Fig. S4A, there were almost no changes in the protein levels of PRDX1, PRDX2, PRDX4 and PRDX6 after FAs + MSUc treatment. In contrast, the protein level of PRDX5 decreased; however, the protein level of PRDX3 increased. These data suggest that Prdx5 and Prdx3 may play important roles in FAs + MSU crystal-induced inflammation. We further confirmed the effect of MaR1 on PRDX3 protein levels. MaR1 had little effect on the elevated PRDX3 protein expression induced by MSUc (Fig. 4C).

Next, we explored whether MaR1 affects Prdx5 expression at the mRNA level. Consistent with the Western blot data, MaR1 significantly inhibited FAs + MSU crystal-induced downregulation of Prdx5 at the mRNA level (Fig. 4D). It has been reported that MaR1 can accelerate Nrf2 activity, and the Keap1-Nrf2 signaling axis affects Prdx5 expression at the mRNA level (Graham et al. 2018; Chen et al. 2020). Western blot data showed that MaR1 greatly suppressed the downregulation of p-NRF2 (assessed by phosphorylation of NRF2 (S40)) induced by FAs + MSUc (Fig. 4E). Immunofluorescence data further showed that MaR1 significantly promoted Nrf2 nuclear translocation in BMDMs treated with FAs + MSUc (Fig. 4F). ML385, a specific inhibitor of Nrf2, significantly reversed the upregulation of Prdx5 mRNA and protein expression induced by MaR1 (Fig. 4G).

**Fig. 5:**
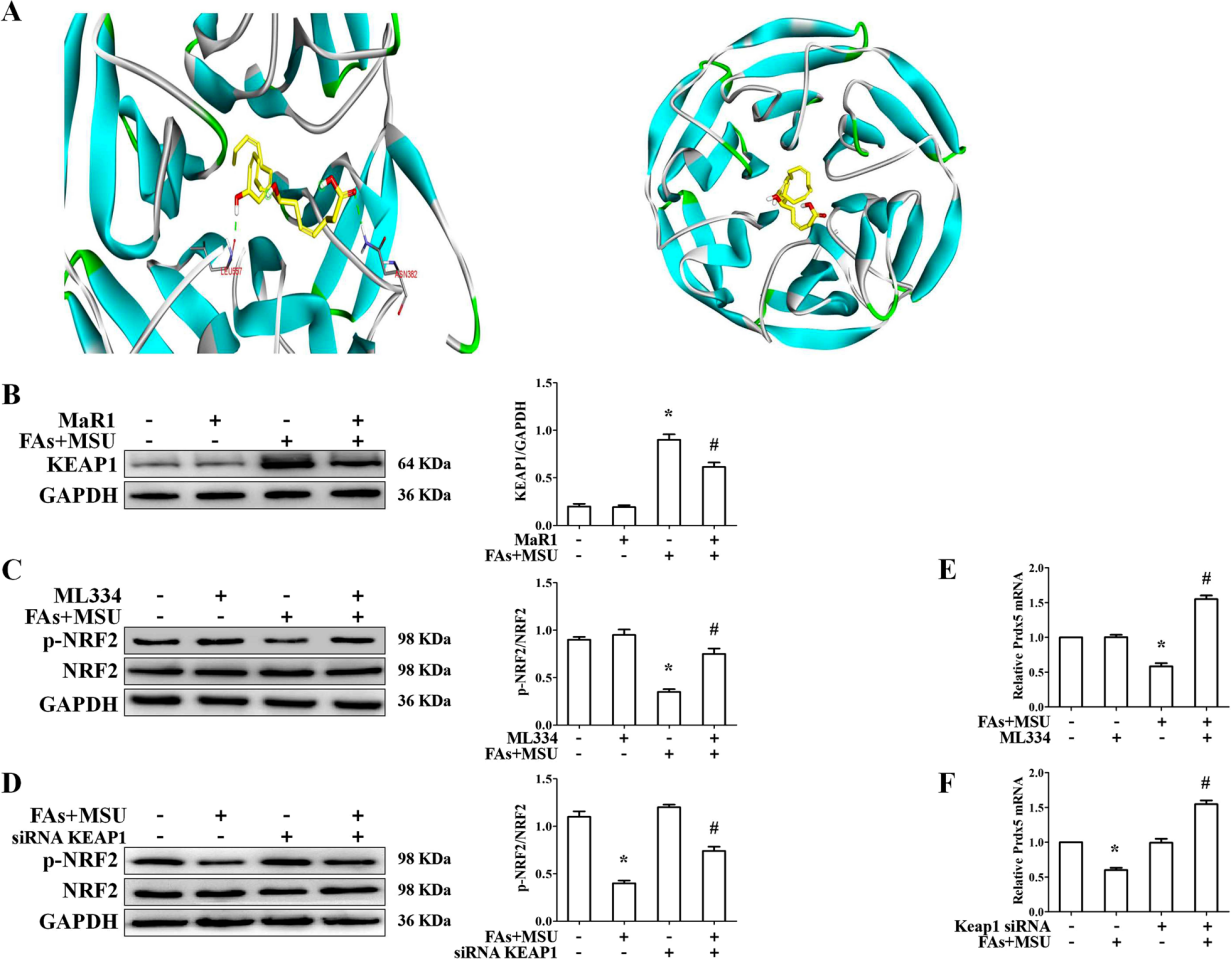
MaR1 regulates the Keap1-Nrf2signaling axis. **A** The docking mode of maresin1 with keap1 (PDB code: 5CGJ). Maresin1 and the amino acid residues that participate in the interactions were displayed as sticks and colored by the element, hydrogen bonds are showed as green dashed lines. **B** Western blot detection of KEAP1 protein level. **C** BMDMs were treated with or without ML334 (1 μM) for 1 h, then stimulated with FAs + MSUc for 12 h. Western blot detection of p-NRF2 protein level. **D** Effect of Keap1 knockdown on p-NRF2 protein level. **E** and **F** ML334 treatment or Keap1 knockdown increased Prdx5 mRNA expression. *Compared with no FAs + MSUc treatment, # compared with FAs + MSUc treatment. * and # means P < 0.05

We wanted to know if MaR1 regulates Prdx5 expression via the Keap1-Nrf2 signaling axis. To further explore the interacting mode of Keap1 with MaR1, molecular docking study, based on the crystal structures of Keap1 (PDB code: 5CGJ), was performed by using the software. The results were shown in Fig. 5A. Finally, the calculated binding energy of MaR1 with Keap1 and Ki value were respectively was -6.85 kcal/mol and 9.55 nM. MaR1 repressed FAs + MSU crystal-induced Keap1 protein expression (Fig. 5B). Both Keap1 knockdown and the inhibitor of Keap1-Nrf2 axis (ML334) could accelerate p-NRF2 protein level in BMDMs treated with FAs + MSUc (Fig. 5C, D and Additional file 1: Fig. S4B). Consistent with this data, both Keap1 knockdown and the inhibitor of Keap1-Nrf2 axis obviously promoted Prdx5 mRNA level (Fig. 5E, )F). These data suggest that MaR1 may accelerate Prdx5 expression through the Keap1-Nrf2 axis in FAs + MSU crystal-induced inflammation.

### Prdx5 accelerated AMPK activity and improved mitochondrial fragmentation

**Fig. 6:**
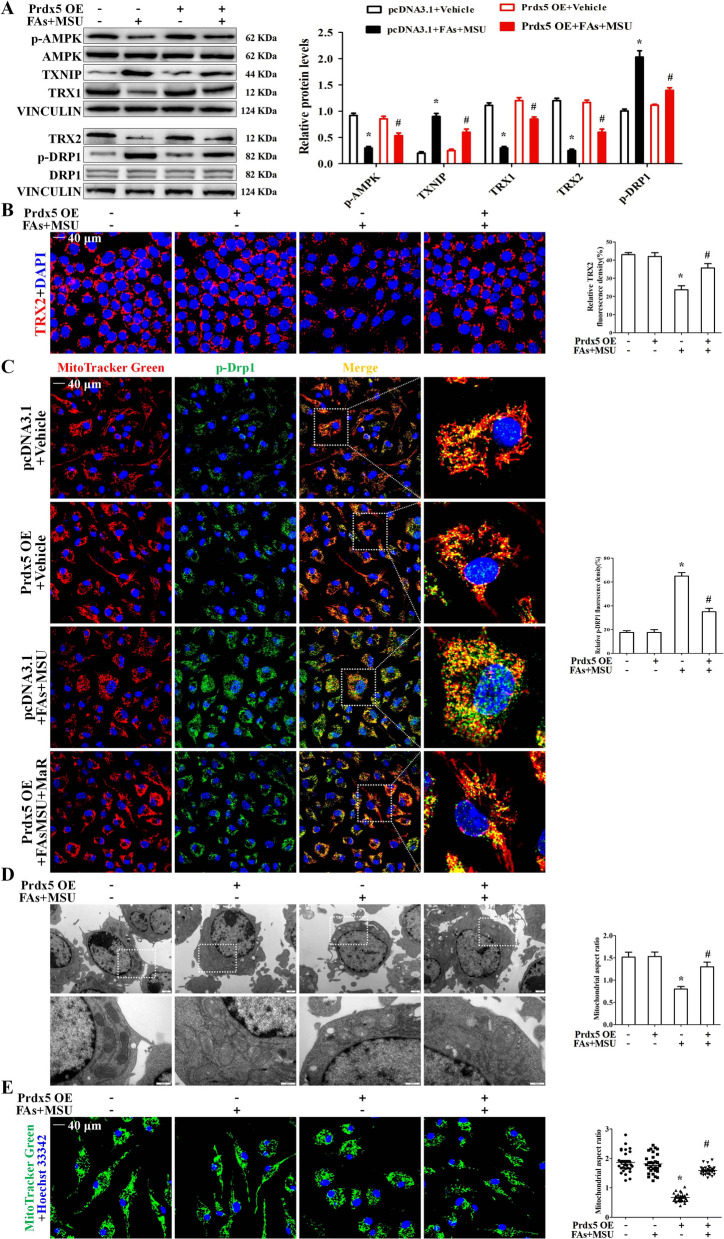
Prdx5 over-expression promotes AMPK activity. BMDMs were transfected with control (pcDNA3.1) or Prdx5 open reading frame (ORF) was cloned into pcDNA3.1 plasmids (Prdx5 OE) for 24 h and then stimulated with FAs + MSUc for 12 h. **A** Western blot analysis for p-AMPK, TXNIP, TRX1, TRX2 and p-DRP1 protein levels. **B** Immunofluorescence staining to detect the signal intensity of TRX2 protein. **C** BMDMs were stained with Mitotracker Red probe, then immunofluorescence staining for p-Drp1 (green). Scale bar: 40 μm. Areas outlined in white are shown enlarged in the right panels. Image is representative of 5 images/dish; n = 3 dishes/condition. **D** Representative TEM images of morphological changes in mitochondria (white box) in BMDMs. Scale bar: 1 μm. Image is representative of 10 images/sample; n = 3 samples/condition. **E** BMDMs were stained with Mitotracker Green probe and DAPI, and mitochondrial morphology was analyzed by LCM imaging. Image is representative of 5 images/dish; n = 3 dishes/condition. *Compared with no FAs + MSUc treatment, # compared with FAs + MSUc treatment. * and # means P < 0.05

A previous study showed that MSUc stimulation inhibits AMPK activity (as indicated by the phosphorylation of AMPKα and p-AMPK) (Wang et al. 2016) and that Prx5 overexpression induces the phosphorylation of AMPKα (Kim et al. 2020). We sought to investigate whether Prdx5 affects AMPKα phosphorylation during FAs + MSUc induced inflammation. The data indicated that Prdx5 overexpression reversed the reduction in AMPK phosphorylation induced by FAs + MSUc (Fig. 6A and Additional file 1: Fig. S5A). Since it has been shown that AMPK activation regulates the TXNIP/TRX balance (McWherter et al. 2018), we next explored the effects of Prdx5 overexpression on the expression of TRX isoforms and TXNIP. As shown in Fig. 6A, FAs + MSUc downregulated the expression of TRX1 and TRX2 isoforms while stimulating the expression of TXNIP. These effects were blocked by Prdx5 overexpression, indicating an ability of Prdx5 to maintain the balance between TRX isoforms and TXNIP. Immunofluorescence data all revealed that Prdx5 overexpression accelerated TRX2 expression in BMDMs treated with FAs + MSUc (Fig. 6B). Our data suggest that compound C, an AMPK inhibitor, hinders the effect of Prdx5 overexpression on TXNIP, TRX1 and TRX2 expression (Additional file 1: Fig. S5B).

A previous study revealed that an AMPK activator blocked Drp1-mediated mitochondrial fission (Wang et al. 2017). We further determined the effect of Prdx5 overexpression on Drp1 activity and the mitochondrial ultrastructure. Prdx5 overexpression repressed Drp1 activity, as reflecting by reduced Drp1 (s616) phosphorylation (Fig. 6A) and Drp1 recruitment to mitochondria (Fig. 6C). This study showed that compound C could block the effect of Prdx5 overexpression on Drp1 activity (Additional file 1: Fig. S5B). Ultrastructure analysis performed by transmission electron microscopy (TEM) indicated that in FAs + MSUc induced BMDMS, the integrity of the mitochondrial cristae is severely disrupted (Fig. 6D). We analyzed the size of mitochondria by measuring the mitochondrial aspect ratio (AR). BMDMs treated with FAs + MSUc exhibited a significant decrease in the AR ratio, which was markedly reversed by Prdx5 overexpression (Fig. 6D), reflecting an improvement in mitochondrial morphology. Mitochondrial morphology in BMDMs stained with MitoTracker Green was further analyzed by laser confocal microscopy (LCM). In BMDMs that were not subjected to FAs + MSUc treatment, most mitochondria formed interconnected, tubular networks (Fig. 6E). In BMDMs treated with FAs + MSUc, the mitochondria were fragmented into small pieces in parallel with mitochondrial network breakdown (Fig. 6E). Prdx5 overexpression significantly inhibited mitochondrial fragmentation (Fig. 6E). However, in Prdx5-deficient BMDMs, MaR1 almost completely lost its ability to affect the expression of p-AMPK and its downstream targets (Additional file 1: Fig. S5C, D).

### Prdx5 relieved impaired fatty acid oxidation induced by FAs + MSUc

**Fig. 7:**
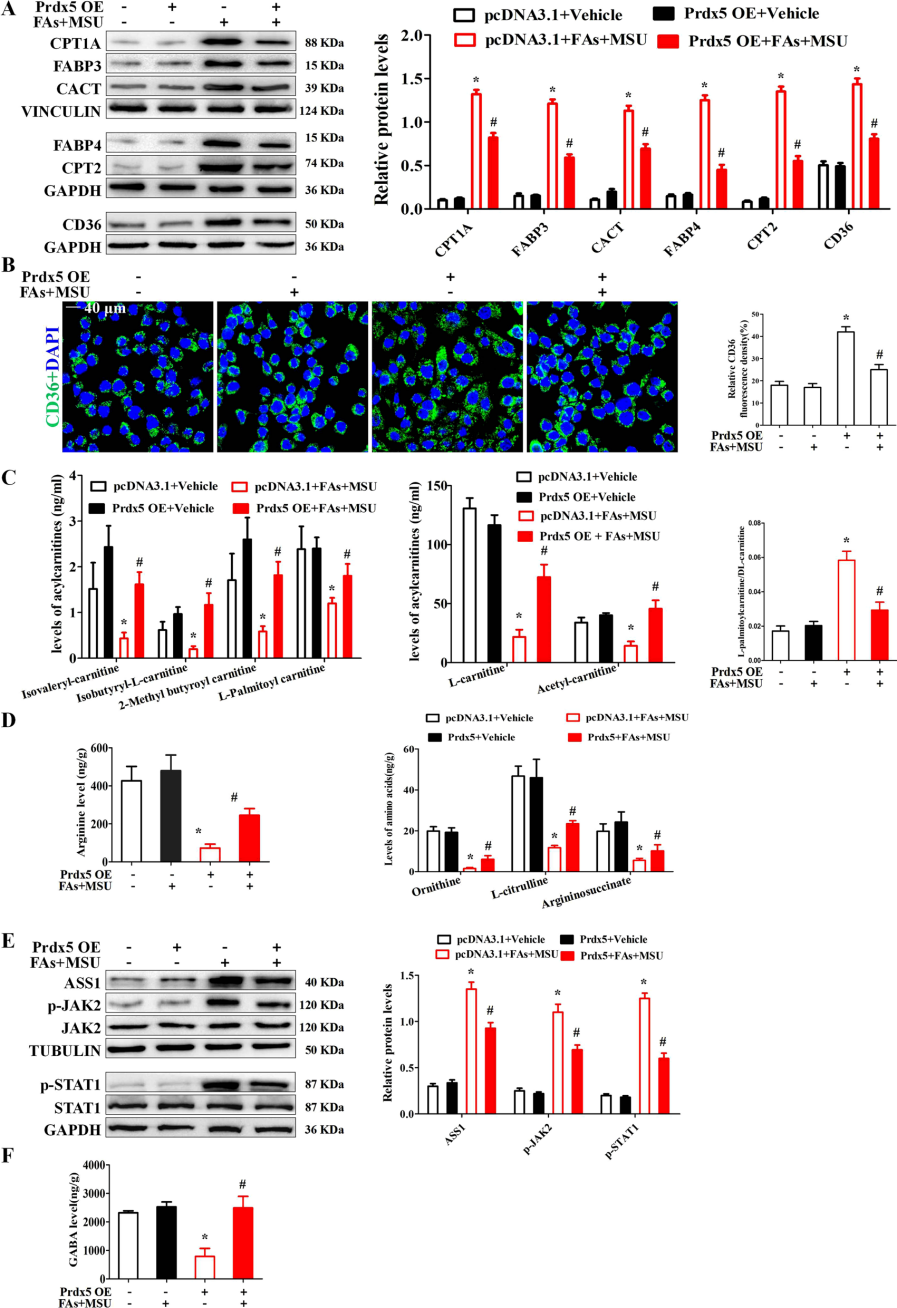
Prdx5 overexpression ameliorates impaired fatty acid oxidation (FAO). **A** BMDMs were transfected control (pcDNA3.1) or pcDNA3.1 inserted into the Prdx5 ORF for 24 h and then stimulated with FAs + MSUc for 12 h. Western blot detection of CPT1A, FABP3, CACT, FABP4, CPT2 and CD36 protein levels. **B** Immunofluorescence staining for CD36. Scale bar: 40 μm. **C** Non-targeted metabolomics (T500) detection and analysis of metabolite levels (Isovaleryl-carnitine, Isobutyryl-L-carnitine, 2-Methylbutyroylcarnitine, L-palmitoylcarnitine, L-carnitine, Acetyl-carnitine). **D** Levels of some metabolites (Ornithine, Citrulline, Argininosuccinate and Arginine) in the Urea cycle. **E** Levels of GABA. **F** Western blot for ASS1, p-JAK2, p-STAT1, JAK2 and STAT1 protein levels. * Compared with no FAs + MSUc treatment, # compared with FAs + MSUc treatment. * and # means P < 0.05

It has been reported that Prdx5 regulates fatty acid oxidation (Kim et al. 2020). Fatty acid binding proteins (FABP) 3 and (FABP) 4 are responsible for intracellular shuttling of fatty acids. FABP3 is closely related to fatty acid oxidation (FAO) (Wu et al. 2022). CD36 is a scavenger receptor mediating long-chain fatty acid (FA) uptake (Guerrero et al. 2022). ACSL5 can activate saturated FAs (SFAs) and convert into fatty acyl-CoA esters, especially for palmitic acid (PA, C16:0). Therefore, we wondered whether FAs + MSUc would affect the expression of fatty acid transporters (FATs) and FAO-related enzymes. FAs or MSUc alone could upregulate the expression of FATs (including CD36, FABP3 and FABP4) and FAO-related enzymes (including CPT1A, CPT2 and CACT) to a certain extent, but the combination of FAs and MSUc synergistically increased the expression of these proteins (Fig. S6A). Next, we further investigated the effect of Prdx5 overexpression on the expression of FATs and FAO-related enzymes. Western blot and RT-qPCR analysis indicated that Prdx5 overexpression reduced the expression of FATs and FAO-related enzymes stimulated by FAs + MSUc (Fig. 7A and Additional file 1: Fig. S6B). Immunofluorescence data showed that FAs + MSUc also promoted CD36 expression, and Prdx5 overexpression significantly inhibited CD36 expression induced by FAs + MSUc (Fig. 7B). We further explored whether MaR1 inhibited FAs + MSU crystal-stimulated expression of FATs and FAO-related enzymes through upregulation of the Prdx5 protein. Similar to Prdx5 overexpression, MaR1 treatment attenuated the expression of FATs and FAO-related enzymes induced by FAs and MSUc (Additional file 1: Fig. S7). In Prdx5-deficient macrophages, MaR1 had almost no effect on the FAs + MSU crystal-induced protein expression of FATs and FAO-related enzymes (Additional file 1: Fig. S7). These data suggest that Prdx5 plays a key role in the regulation of fatty acid oxidation by MaR1.

Since Prdx5 affects the expression of FATs and key enzymes for FAO, we further explored the effect of Prdx5 overexpression on metabolites in BMDMs treated with MSUc. A total of 269 metabolites were evaluated with the T500 untargeted metabolomics assay. There were 43 metabolites that were significantly different between pcDNA3.1 + Vehicle vs pcDNA3.1 + FAs MSU and pcDNA3.1 (FAs MSU) vs Prdx5 overexpression (FAs MSU), and the heat-maps are shown in Additional file 1: Figs. S8 and S9. Acylcarnitine levels are closely related to fatty acid oxidation. MaR1 reversed the decrease in short-chain acylcarnitine levels (including Isovaleryl-carnitine, Isobutyryl-L-carnitine, 2-Methylbutyroylcarnitine, L-carnitine, Acetyl-carnitine) and long-chain acylcarnitine (L-palmitoylcarnitine) (Fig. 7C). MaR1 reduced the elevated ratio of L-palmitoylcarnitine to L-carnitine induced by FAs + MSUc (Fig. 7C), suggesting that MaR1 inhibited the activity of CPT1A, which is the limiting enzyme of FAO. As shown in Additional file 1: Fig. S10A, FAs + MSUc stimulation resulted in a significant decrease in the levels of lysine and methionine which are the precursors for carnitine biosynthesis, but MaR1 treatment greatly upregulated the levels of Lysine and L-Methionine (Additional file 1: Fig. S10A). FAO is associated with ATP production, and we further explored the effect of MaR1 on ATP levels. MaR1 also blocked the decrease in ATP levels induced by FAs + MSUc (Additional file 1: Fig. S10B). In response to FAs + MSUc, there was a decrease in the levels of intermediate metabolites (including Ornithine, L-citrulline, Argininosuccinate and Arginine) in the urea cycle (Fig. 7D), while MaR1 treatment reversed this phenomenon (Fig. 7D). JAK2-STAT1 signaling and ASS1 expression levels have been reported to correlate with the urea cycle (Mao et al. 2022), and Prdx5 overexpression inhibited the JAK2-STAT1 signaling and ASS1 protein expression induced by FAs + MSUc (Fig. 7E). A recent study has reported that GABA inhibits NLRP3 inflammasome activation and IL-1β secretion in macrophages (Fu et al. 2022). Our metabolomics data showed that Prdx5 overexpression significantly upregulated GABA levels in BMDMs treated with FAs + MSUc (Fig. 7F).

### MaR1 and Prdx5 are involved in MSU crystal-induced inflammation in vivo

**Fig. 8:**
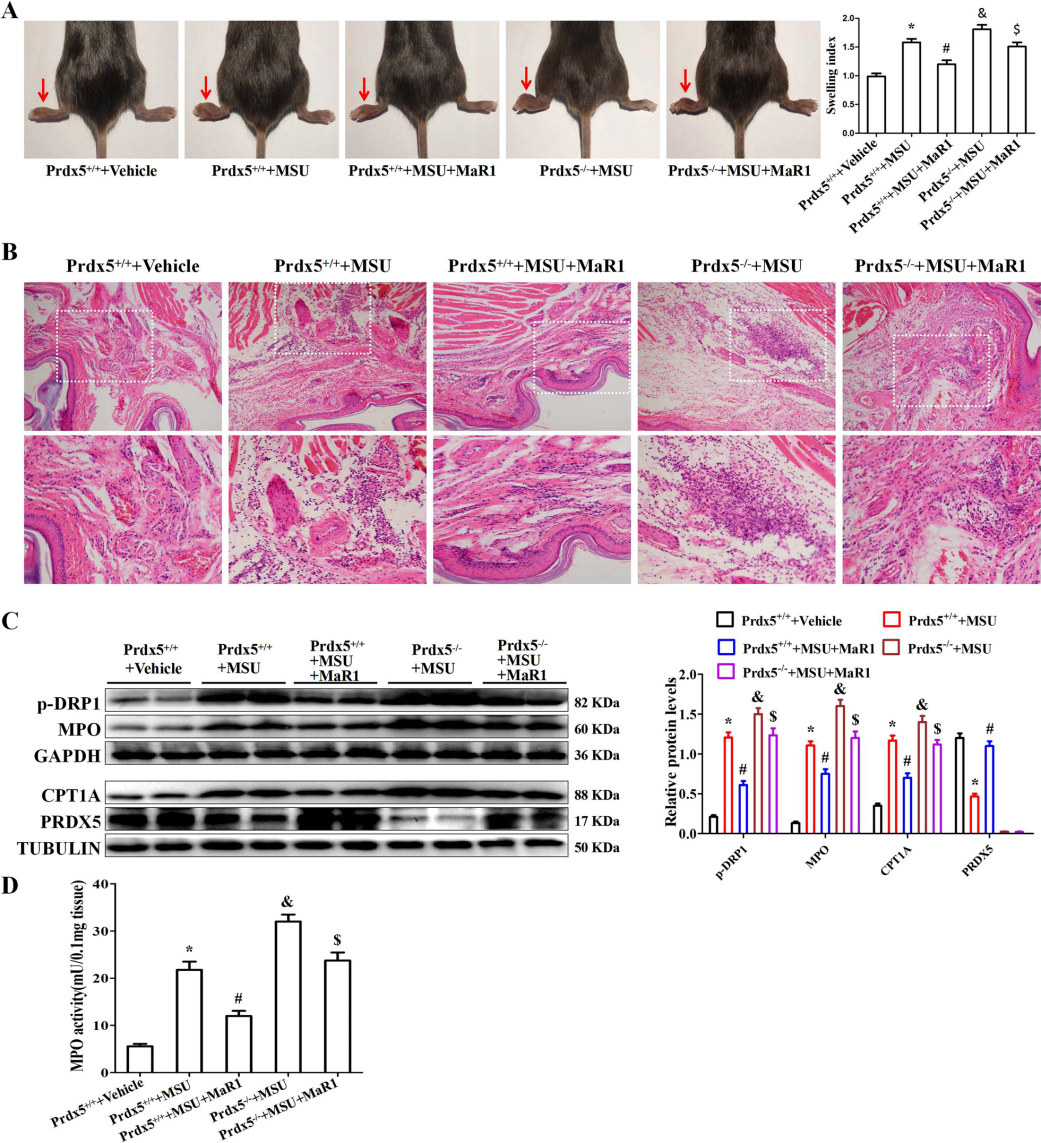
MaR1 inhibits MSUc induced joint inflammation in a mouse model. **A** Detection and analysis of swelling index of mouse foot-pad. **B** Detection of immune cell infiltration in mouse foot-pad tissue sections by hematoxylin–eosin (HE) staining. **C** Western blot detection of p-DRP1, MPO, CPT1A and PRDX5 protein levels in mouse foot-pad tissue. **D** Detection of MPO activity in mouse foot-pad tissue lysate. *Compared with Prdx5^+/+^  + Vehicle, # compared with Prdx5^+/+^  + MSUc, & compared with Prdx5^+/+^  + MSUc, $ compared with Prdx5^+/+^  + MSUc + MaR1. *, #, & and $ means P < 0.05. N = 5 each group

Based on the anti-inflammatory function of MaR1 and Prdx5 in vitro, we investigated whether MaR1 treatment or Prdx5 deficiency influenced the severity of MSU crystal-induced gouty arthritis in vivo. A model of MSU crystal-induced swelling of mouse foot-pad was used to assess the severity of inflammation. After MSU crystal challenge, MaR1 treatment inhibited the increase in the swelling index in WT mice (Fig. 8A). Compared to WT mice injected with MSUc, Prdx5-deficient mice showed an increase in the swelling index, and MaR1 treatment did not improve the swelling index in Prdx5-deficient mice (Fig. 8A). As shown in Fig. 8B, MaR1 treatment inhibited immune cell infiltration, whereas Prdx5 deficiency exacerbated inflammatory cell infiltration (Fig. 8B). Western blot data indicated that MSUc injection into mouse foot-pad tissue resulted in elevated MPO, CPT1A and p-DRP1 protein expression, while PRDX5 protein expression was reduced. In Prdx5^+/+^ mice injected with MSUc, MaR1 treatment inhibited MPO, CPT1A and p-DRP1 protein expression while promoting PRDX5 protein expression (Fig. 8C). The myeloperoxidase (MPO) activity in a homogenate of foot-pad tissues was analyzed to investigate the recruitment of neutrophils induced by MSUc. MaR1 effectively decreased the MPO activity induced by MSUc (Fig. 8D). Immunofluorescence staining indicated that MaR1 blocked the distribution of CD68-, MPO-, Ly6G- and CPT1A-positive cells in foot-pad tissue sections (Additional file 1: Fig. S11).

**Fig. 9:**
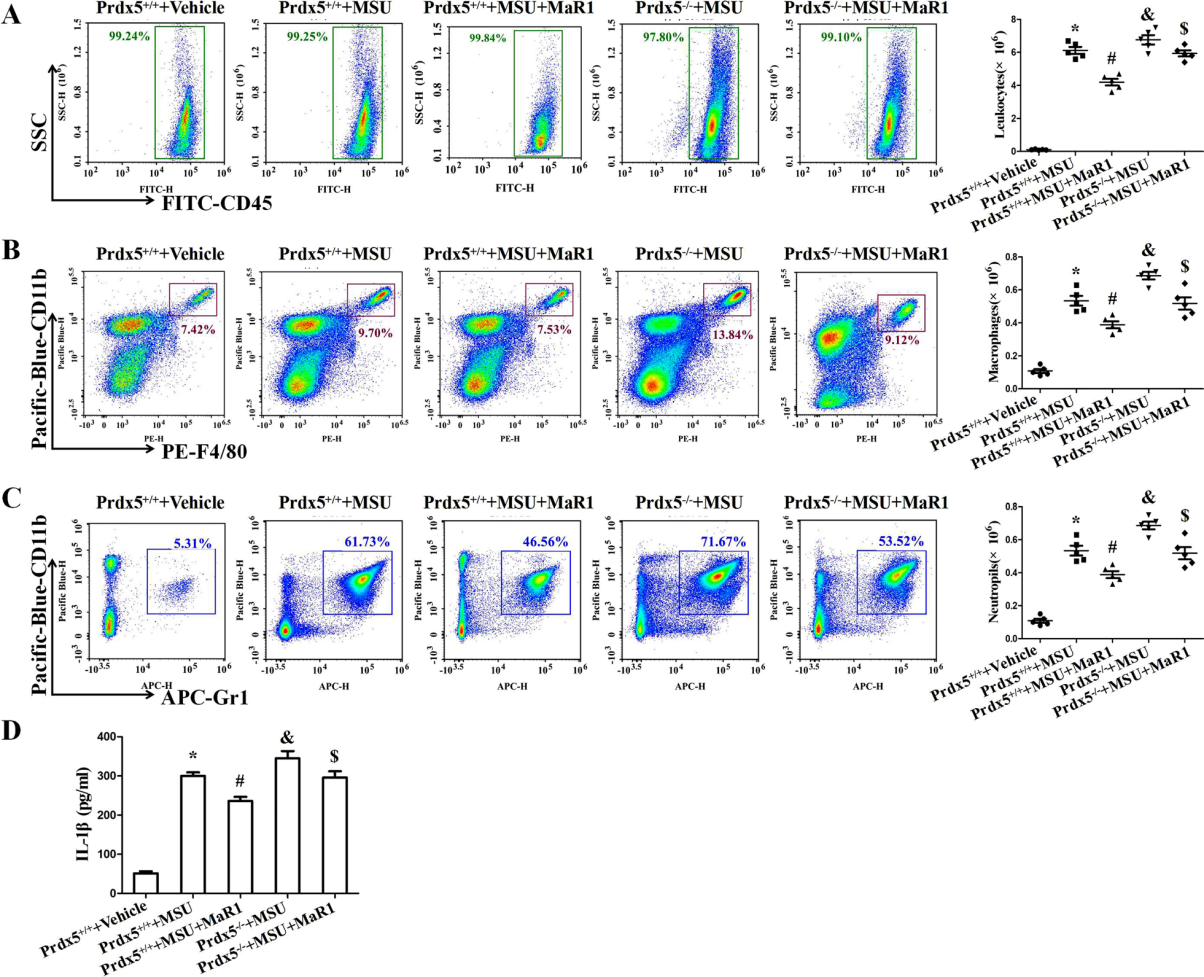
MaR1 decreases MSUc induced peritoneal inflammation in a mouse model. **A–C** Detection of leukocytes (CD45), macrophages (F4/80), neutrophils (Gr-1) in peritoneal fluid by FCM. **D** ELISA for IL-1β levels in peritoneal fluid. N = 5 each group. *Compared with Prdx5^+/+^  + Vehicle, # compared with Prdx5^+/+^  + MSUc, & compared with Prdx5^+/+^  + MSUc, $ compared with Prdx5^+/+^  + MSUc + MaR1. *, #, & and $ means P < 0.05

We further explored the function of MaR1 and Prdx5 in a model of MSU crystal-induced peritonitis. In comparison with that of WT mice injected with MSUc, the number of leukocytes in the peritoneal lavage fluid (PCLF) of mice treated with MaR1 was significantly reduced after MSU crystal challenge (Fig. 9A). MaR1 also reduced the infiltration of macrophages (CD11b^+^ F4/80^+^) and neutrophils (CD11b^+^ Gr1^+^) into the peritoneal cavity induced by MSUc (Fig. 9B and Fig. 9C). In WT mice injected with MaR1 + MSUc, MaR1 treatment also greatly attenuated IL-1β secretion in the peritoneal lavage fluid compared to WT mice injected with MSUc (Fig. 9D).

## Discussion

MaR1 is a new member of the family of proresolving mediators derived from endogenous DHA. It has been demonstrated that MaR1 plays an important protective role in inflammatory diseases. In this study, our findings indicated that MaR1 suppressed the expression of key genes for MSU crystal-induced inflammation in vitro. In vivo, MaR1 attenuated the swelling associated with MSU crystal-induced gouty arthritis and inflammatory cell migration in the peritoneal cavity. MaR1 has been shown to regulate ROS generation and improve mitochondrial function (Gu et al. 2018; Calderin et al. 2022; Li et al. 2021). Our data revealed that MaR1 not only reduced mitochondrial ROS levels and reverses mitochondrial membrane depolarization but also inhibited mPTP opening and [Ca^2+^] m changes. Mitochondrial ROS react with newly synthesized mtDNA to produce oxidized mtDNA (Ox-mtDNA) (Dalbeth et al. 2016). MaR1 effectively blunted mtDNA synthesis and blocked the generation of cytoplasmic Ox-mtDNA induced by FAs + MSUc. It has been reported that cytoplasmic Ox-mtDNA promotes NLRP3 inflammasome activation (Xian et al. 2022). These data suggest that MaR1 plays an important role in ameliorating the mitochondrial dysfunction induced by FAs + MSUc to inhibit NLRP3 inflammasome activation.

MSUc stimulation of macrophages or MSUc injection into mouse foot pads both resulted in decreased Prdx5 protein expression, and MaR1 treatment effectively increased Prdx5 protein levels. Notably, the inhibitory effect of MaR1 on MSU crystal-induced inflammation is diminished when Prdx5 is defective. Our findings suggest that Prdx5 plays a crucial role in the inhibition of MSU crystal-induced inflammation by MaR1. A previous study has confirmed that Keap1-Nrf2 axis regulates Prdx5 expression and Prdx5 is an important anti-inflammatory Nrf2 target gene (Graham et al. 2018). The docking study and Western blot data indicated that MaR1 efficiently removed the inhibition of Nrf2 activity by Keap1, strongly supporting that MaR1 may promote Prdx5 expression by regulating the Keap1-Nrf2 signaling axis.

It has been demonstrated that Prx5 promotes AMPK activity (Kim et al. 2020) and AMPK activation can suppress TXNIP expression (Liu et al. 2022). TXNIP has the ability to bind to thioredoxin (TRX) and inhibit the function and expression of TRX. Our data are consistent with previous reports that MSUc disrupted the balance of TRX and TXNIP proteins, reflecting that MSUc promoted the expression of TRX1 and TRX2 isoforms and decreased TXNIP protein levels. These effects were reversed by MaR1, which means that MaR1 has the ability to protect the balance between the TRX isoforms and TXNIP. An AMPK inhibitor (compound C) prevented Prdx5 from regulating the balance between TXNIP and TRX isoforms. This suggests that Prdx5 may regulate the balance of TRX and TXNIP proteins by promoting AMPK activity. Loss of TRX2 induces mitochondrial ROS over-production, mitochondrial integrity disruption and cytosolic release of mitochondrial DNA which in turn activate the NLRP3 inflammasome pathways (Huang et al. 2022). AMPK activation inhibits Drp1-mediated mitochondrial fission (Wang et al. 2017). Drp1 activity is associated with NLRP3 inflammasome activation (Rong et al. 2022). In FAs + MSU crystal-induced inflammation, MaR1 treatment or Prdx5 overexpression reduced p-Drp1 (s616) levels by upregulating AMPK activity. AMPK functions as an inhibitor of NF-κB and the NLRP3 inflammasome, promotes the polarization of anti-inflammatory macrophages, and significantly reduces the inflammatory response of cultured macrophages to MSUc (Wang et al. 2016). These data strongly suggest that MaR1 may inhibit NLRP3 inflammasome activation via Prdx5/AMPK signaling.

Prdx5 can regulate fatty acid metabolism and recent study has revealed that there exists significant linear relationship between mitochondrial fragmentation and FAO rate (Ngo et al. 2023). It has been confirmed that blocking FAO in macrophages alleviates FAs + MSU crystal-induced inflammation (Hall et al. 2018). FAO is a complex process that depends on several biochemical steps and enzymatic systems. CD36 or FABPs transport long-chain FAs into intracellular. Intracellular long-chain FAs is esterified to acyl-CoA by enzymes of the long-chain acyl-CoA synthase (ACSL) family, which is then conjugated to carnitine by carnitine palmitoyltransferase 1 (CPT1) to convert it to acyl-carnitine. After mitochondrial translocation via carnitine acylcarnitine translocase (CACT), acylcarnitine is converted back to acyl-CoA by CPT2 and then enters the fatty acid β-oxidation (FAO) pathway, followed by the mitochondrial tricarboxylic acid (TCA) cycle (Fujiwara et al. 2018). The present study revealed that Prdx5 can effectively inhibit the expression of long-chain FAs transporters and FAO-related enzymes, but the exact molecular mechanisms by which Prdx5 inhibits the expression of these proteins need to be further investigated.

One of the main functions of L-carnitine is to shuttle long-chain FAs from the cell membrane into the mitochondrial matrix for FAO. This study showed that L-carnitine levels were significantly decreased in BMDMs treated with FAs + MSUc. But FAs + MSUc stimulation accelerated the expression of long-chain FAs transporters and FAO-related enzymes, especially CPT1A. During this process of FAO, CPT1A is rate limiting enzyme because it plays a key role in transporting medium-length acyl-CoA chains to the mitochondrial matrix along with L-carnitine (Schlaepfer and Joshi 2020). In response to FAs + MSUc, the ratio of L-palmitoylcarnitine to L-carnitine is increased, reflecting the acceleration of FAO by FAs + MSUc. These effects were blocked by Prdx5 overexpression, indicating that Prdx5 ameliorates impaired FAO induced by FAs + MSUc. Further studies are required to explore how Prdx5 promotes intracellular levels of L-carnitine and whether carnitine levels correlate with the expression of FAO-related protein.

In addition, we unexpectedly found that macrophage L-citrulline levels declined rapidly in response to FAs + MSUc. ASS1 functions as a pro-inflammatory regulator that converts citrulline to argininosuccinate (Orecchioni et al. 2020). ASS1 can deplete citrulline rapidly and efficiently and serves as a metabolic checkpoint for the innate immune response (Rong et al. 2022). Our data are consistent with the previous notion that ASS1 expression is upregulated during inflammatory macrophage activation (Nussler et al. 1994). Prdx5 overexpression significantly decreased ASS1 protein expression and increased the levels of urea cycle intermediate metabolites, suggesting that Prdx5 overexpression promotes the urea cycle. It has been reported that activation of JAK2-STAT1 signaling requires ASS1 to deplete citrulline (Rong et al. 2022). Our data showed that Prdx5 overexpression effectively inhibited JAK2-STAT1 signaling. Whether Prdx5 directly affects ASS1 expression or promotes ASS1 expression at the transcriptional level via JAK2-STAT1 signaling remains unclear.

## Conclusions

MaR1 exerts anti-inflammatory effects on MSU crystal-induced inflammation in vitro and in vivo, promoting Prdx5 expression mainly through regulation of the Keap1-Nrf2 signaling axis. Prdx5 protects mitochondrial function by activating AMPK and its downstream signaling pathways. In particular, MaR1 also improved FA + MSU crystal-induced FAO impairment. These data suggest that MaR1 and Prdx5 may be potential targets for gout therapy in the future.

### Supplementary Information

**Additional file 1: Table S1.** The antibodies were used in Western blotting immunofluorescence assays.**Table S2.** The targeted siRNA sequence for mice Keap1. **Table S3.** The sequences of primer for PCR amplification.**Table S4.** The information about the genes encoded by the DEPs. **Fig. S1. **MaR1 inhibits the secretion of IL-1β, IL-6, TNF-α. ELISA assay was used to detect IL-1β, IL-6, TNF-α, TGF-β and IL-10 levels in cell culture supernatants. * compared with no FAs + MSU crystals treatment, # compared with FAs + MSUc treatment. * and # means P<0.05. **Fig. S2.** Relative total mtDNA amounts in BMDMs treated with or without MaR1 for 1 h, followed by FAs + MSUc for 12 h. Shown are the ratio of D-loop mtDNA to Tert nuclear (n) DNA, Cox1 mtDNA to 18S nDNA or mtDNA that is not inserted into nuclear DNA (non-NUMT) to B_2_m nDNA.* compared with no FAs + MSUc treatment, # compared with FAs + MSUc treatment. * and # means P<0.05. **Fig. S3.** Expression analysis of differentially expressed proteins (DEPs) in BMDMs treated with or without MaR1 for 1 h, followed by FAs + MSUc for 12 h. Heat-map was used to show the DEGs. The colors ranging from red to blue indicate the protein levels from high to low. MSU-1, MSU-2 and MSU-3 represent FAs + MSUc treatment groups, MaR-1, MaR-2 and MaR-3 represent MaR1 and FAs + MSUc treatment groups. N=3 each group. **Fig. S4.** Effect of FAs, MSU crystals, FAs + MSUc stimulation on PRDX1-6 protein levels in BMDMs and targeting Keap1 siRNA to inhibit KEAP1 protein expression. (A)BMDMs were treated with FAs, MSUc  or FAs + MSUc for 12 h. Western blot detection of PRDX1-6 protein levels. * compared with Vehicle (BSA) treatment. (**B)**Western blot detection of KEAP1 protein level. *compared to BMDMs transfected with negative control siRNAs. * means P<0.05. **Fig. S5.** PRDX5 protein expression and Compound C reversed the effect of Prdx5 overexpression on AMPK activation and downstream target proteins. (A)BMDMs were transfected with pcDNA3.1 or Prdx5 ORF was cloned into pcDNA3.1 plasmid. Western blot for PRDX5 protein level. *compared with transfection pcDNA3.1 plasmid. (B)Western blot for p-AMPK, TXNIP, TRX1,TRX2 and p-DRP1protein levels.*compared with pcDNA3.1 + FAs + MSU crystals.# compared with Prdx5 OE + FAs + MSUc. (C) Detection of PRDX5 protein level in BMDMs derived from Prdx5^+/+^ and Prdx5^-/-^ mice.*compared with BMDMs derived from Prdx5^+/+^. * and # means P<0.05. (D) BMDMs from Prdx5^+/+^ and Prdx5^-/-^ mice were treated with or without MaR1, then stimulated with FAs + MSUc. Western blot for p-AMPK, TXNIP, TRX1, TRX2 and p-DRP1 protein levels.*compared with Prdx5^+/+^ + FAs + MSUc. # compared with Prdx5^+/+^ + FAs + MSU crystals + MaR1. * and # means P<0.05. **Fig. S6.** Effects of FAs, MSUc and FAs + MSU crystals stimulation on the expression of FAO related genes. (A) BMDMs were treated with FAs, MSUc or FAs + MSUc for 12 h. Western blot detection of FABP4, CPT1A, FABP3, CACT, CPT2 and CD36 protein levels. * compared with no FAs, MSUc and FAs + MSUc treatment. * means P<0.05. (B) Quantitative PCR to detect the mRNA level of ACSL5. * compared with no FAs + MSU crystals treatment,# compared with FAs + MSUc treatment. * and # means P<0.05. **Fig. S7.** MaR1 affects AMPK activity mainly through upregulation Prdx5 expression. BMDMs from Prdx5^+/+^ and Prdx5^-/-^ mice were treated with or without MaR1, then stimulated with FAs + MSUc. Western blot for CPT1A, FABP3,CACT, FABP4 and CPT2 protein levels. *compared with Prdx5^+/+^ + FAs + MSUc. # compared with Prdx5^+/+^ + FAs + MSUc + MaR1. * and # means P<0.05. **Fig. S8.** The heat-map showed the effects of FAs + MSUc on metabolites in BMDMs. **Fig. S9.** The heatmap listed the effects of Prdx5 overexpression on metabolites in BMDMs treated with FAs + MSUc. **Fig. S10.** Prdx5 overexpression increases Lysine, L-Methionine and ATP production. (A) The levels of Lysine and L-Methionine.  (B) The levels of ATP.*compared with Vehicle (BSA) treatment, # compared with pcDNA3.1 + FAs + MSUc. **Fig. S11.** MaR1 treatment inhibited the distribution of CD68, Ly6G, MPO and CPT1A proteins induced by MSU crystals in mice footpad tissue section. Immunofluorescence detection of CD68, Ly6G, MPO and CPT1A protein distribution. *compared with Prdx5^+/+^ + MSU, # compared with Prdx5^+/+^ + MSU, & compared with Prdx5^+/+^ + MSU, $ compared with Prdx5^+/+^ + MSU + MaR1. *, # and & means P<0.05.

## Data Availability

The data used in this study are available upon request.
