# Supplementary material for: Maresin1 ameliorates MSU crystal-induced inflammation by upregulating Prdx5 expression
Source: Mol Med. 2023 Nov 23;29:158. doi: 10.1186/s10020-023-00756-w (PMC10668345; doi:10.1186/s10020-023-00756-w)

[**Supplementary Material**](file:///E:\#MOESM1)**s and Methods**

**Preparation of palmitic and stearic acids**

Stock Palmitic (C16:0; Sigma-Aldrich) and stearic (C18:0; Sigma-Aldrich) acids were dissolved at 90°C in 100 mM NaOH to a concentration of 50 mM, and then diluted 1:5 with a 5% solution of BSA at 37°C. BSA-conjugated FAs were then filtered through a 0.2-μm Acrodisc syringe filter and stored at - 20°C.

**Detailed experimental methods for DIA- proteomic sequencing of BMDMs**

BMDMs were treated with or without MaR1 for 1 h, then stimulated with FAs + MSU crystals for 12 h. Cells were collected and then added SDT buffer (4% SDS, 100 mM Tris-HCl, pH7.6). The lysates were further sonicated, and boiled for 15 min. After centrifuged at 14000 g for 40 min, the supernatant was quantified with the BCA Protein Assay Kit (Bio-Rad, USA). Aliquots of each sample were mixed into one sample for quality control and data-independent acquisition (DIA) library construction. Protein digestion was implemented in accordance with the filter-aided sample preparation procedure. DIA analysis was performed with a high-performance liquid chromatography system Easy nLC-1200 (Thermo Scientific). Buffer A：0.1% formic acid (FA, 06450, Fluka); buffer B：0.1% FA, 84% Acetonirile (ACN，I592230123, Merck). The column was balanced with 95% buffer A. The peptide was first loaded onto an EASY-Spray TM C18 Trap column (Thermo Scientific, P/N 164946, 3 um, 75 um*2 cm), then separated on an EASY-Spray TM C18 LC Analytical Column (Thermo Scientific, ES802, 2 um, 75 um*25 cm) with a linear gradient of buffer B at a flow rate of 250nl /min over 90 min. Liquid separation gradient of buffer B is as follows: 0-40min, the linear gradient is from 8% to 30%; 40-50min, linear gradient is from 30% to 100%; 50-65min, the linear gradient increased to 100% and maintained. The separated samples were analyzed by Q-Exactive HFX mass spectrometer (Thermo Scientific). MS detection method was positive ion, the scan range was 300-1800m/z, resolution for MS1 scan was 60000 at 200 m/z, target of automatic gain control (AGC) was 3e6, maximum injection time (IT) was 50ms. Each full MS–SIM scan followed 20 MS2 scans. Resolution for MS2 scan was 30000, AGC target was 3e6, maximum IT was 120ms and normalized collision energy was 27eV. DIA data were directly imported into Spectronaut software (Spectronaut^TM^ 14.4.200727.47784) to construct a spectral library. The database can be downloaded from the following website: <http://www.uniprot.org>. All of the data reported were based on a protein identification confidence of 99% as defined through a false discovery rate (FDR) ≤ 1%. Each sample was mixed with 2μg iRT standard peptide and DIA mass spectrometry was performed. Each DIA cycle contained one full MS–SIM scan, and 30 DIA scans covered a mass range of 350–1650 m/z with the following settings: SIM full scan resolution was 60,000 at 200 m/z; AGC: 3e6; maximum IT: 50ms. DIA scans were set at a resolution of 30,000; AGC: 3e6; maximum IT: auto; normalized collision energy was 30eV. DIA data were analyzed with Spectronaut searching against the as-established spectral library. All results were filtered based on FDR < 1%. Differentially expressed proteins (DEPs) between two groups were selected with a fold change (FC) greater than 1.2 or lower than 0.67 and a *p* value less than 0.05 (Student’s t test). They were annotated with Gene Ontology (GO) and Kyoto Encyclopedia of Genes and Genomes (KEGG), and further bio-informatic analysis was performed.

**Metabolite profiling analysis**

The sample was thawed on ice, 100 μL of ultrapure water extract was added to resuspend the cell pellet. Divide 50 μL cell suspensions and add 200 µL of methanol (precooled at -20°C) and vortexed for 2 min under the condition of 2500 r/min. The sample was frozen in liquid nitrogen for 5 min, removed on ice for 5 min, after that, the sample was vortexed for 2 min.The previous step was repeated for 3 times. The sample was centrifuged at 12000 rpm/min for 10 min at 4°C. Take 200 μL of supernatant into a new centrifuge tube and place the supernatant in -20°C refrigerator for 30 min. Then the supernatant was centrifuged at 12000 rpm/min for 10 min at 4°C. After centrifugation, transfer 180 μL of supernatant through Protein Precipitation Plate for further liquid Chromatograph Mass Spectrometer (LC-MS) analysis. The left 50 μL cell suspension was frozen and thawed for 3 times, centrifuged at 12,000 r/min for 10 min, and the supernatant was taken to determine the protein concentration by BCA Protein Assay kit.

All those metobolites were detected by MetWare (<http://www.metware.cn/>) based on the AB Sciex QTRAP 6500 LC-MS/MS platform.

**Molecular modeling study of MaR1 and KEAP1**

The KEAP1 protein used for docking was downloaded from the PDB database (PDB code: 5CGJ) and the original ligand RA839 and water molecules were removed. The 3D structures of the small molecules to be tested were constructed and the geometric conformations were optimised by molecular dynamics. The docking studies were performed using the AUTODOCK 4.2 package and the visualisation software Autodock Tools (ADT, version 1.5.6) for the pre-processing of proteins and small molecules and the setting of docking parameters. The pre-processing of the protein and small molecule consisted of (1) adding polar hydrogen to the amino acid residues of the protein and loading each atom of the protein molecule with a Gasteiger charge and (2) performing charge calculations on the small molecule and setting the rotatable bonds. The resulting protein structure is used as input to the AUTOGRID program. Lattice point calculations of atomic affinity were performed for each atom type with AUTOGRID as the ligand, and calculations included charge potential energy and desolation interactions. Each grid point was placed 0.375 Å apart at the bottom of the enzyme activity (coordinates: x = 39.832, y = -20.257, z = -4.736) and the grid size of the grid box was set at 60 Å × 60 Å × 60 Å to include all residues of the activity pocket. Docking was calculated using the Lamarckian genetic algorithm (LGA) with a random population of 100 individuals, an energy evaluation factor of 2500000 and other parameters set as software defaults. Docking results with a root mean square deviation value of 1.0 or less were subjected to cluster analysis, and the optimal cluster docking results for each compound were recorded and analysed, using the binding free energy as an evaluation criterion for docking results. The smaller the binding free energy value, the better the inhibitory activity of the compound. Docking images were generated and observed in Autodock Tools or Discovery Studio 2.5.

**MSU crystal-induced joint inflammation and peritonitis**

To obtain a mouse model of acute joint inflammation, 40μL of sterile saline was injected to the right hind paw as self-control, and 40 μL of MSU crystals (1mg MSU crystals in 40 μL saline) was injected to the left hind paw as an experiment group. At the 24th hour, the thickness of the paws of the mice is analyzed by digital vernier caliper. Then the mice were sacrificed under CO_2_ anesthesia before the paws were extracted.

MSU crystal-induced peritonitis was induced by intraperitoneal injection of 1 mg MSU crystals dissolved in 0.5 ml sterile PBS. After 6 h, mice were killed by exposure to CO2 and peritoneal cavities were washed with 10 ml cold PBS. After centrifugation of the peritoneal lavage, the supernatant was collected to detect IL-1β production by ELISA and the precipitate was stained with the appropriate antibody for FCM analysis.

**Supplementary Tables**

**Table 1 The antibodies were used in Western blotting immunofluorescence assays**

| **Antibodies** | **Source** | **Identifier** | |
| --- | --- | --- | --- |
| Rabbit monoclonal CPT2 | HUABIO | Cat#ET1611-64 |  |
| Rabbit monoclonal CATC | HUABIO | Cat#ER1916-73 |  |
| Rabbit monoclonal FABP4 | HUABIO | Cat#ET1703-98 |  |
| Rabbit monoclonal FABP3 | HUABIO | Cat#ET1706-50 |  |
| Rabbit monoclonal CPT1A | Abcam | Cat#ab234111 |  |
| Rabbit monoclonal p65(Phospho S529) | HUABIO | Cat#ER63592 |  |
| Rabbit monoclonal p65 | HUABIO | Cat#ET1603-12 |  |
| Rabbit monoclonal H3 | HUABIO | Cat#ET1601-30 |  |
| Rabbit monoclonal INOS | Abcam | Cat#ab178945 |  |
| Rabbit monoclonal COX-2 | HUABIO | Cat#ET1610-23 |  |
| Rabbit monoclonal PRDX1 | HUABIO | Cat#ET1702-08 |  |
| Rabbit monoclonal PRDX2 | HUABIO | Cat#ET1502-10 |  |
| Rabbit monoclonal PRDX3 | Bioworld | Cat#PB9349 |  |
| Rabbit polyclonal PRDX4 | Proteintech | Cat#10703-1-AP |  |
| Rabbit monoclonal PRDX5 | Abcam | Cat#ab180587 |  |
| Rabbit polyclonal PRDX6 | Absin | Cat#abs106246 |  |
| Mouse monoclonal Caspase-1 | AdipoGen | Cat#AG-20B-0042 |  |
| Rabbit monoclonal IL-1β | Cell Signaling | Cat#ASP117 |  |
| Rabbit Polyclonal NLRP3 | HUABIO | Cat#ER1706-72 |  |
| Rabbit monoclonal DRP1 | Bioworld | Cat#Bs7390 |  |
| Rabbit monoclonal DRP1(Phospho S16) | Cell Signaling | Cat#3455s |  |
| Rabbit monoclonal AMPKα(Phospho Thr172) | Cell Signaling | Cat#2535 |  |
| Rabbit monoclonal AMPK | HUABIO | Cat#ET1608-40 |  |
| Rabbit monoclonal TRX1 | Abcam | Cat#ab273877 |  |
| Rabbit monoclonal TRX2 | Abcam | Cat#ab185544 |  |
| Rabbit monoclonal TXNIP | HUABIO | Cat#ET1705-94 |  |
| Rabbit monoclonal UCP2 | ZENBIO | Cat#162448 |  |
| Rabbit monoclonal MICU2 | ZENBIO | Cat#824423 |  |
| Rabbit monoclonal CMPK2 | Immunoway | Cat#YT6811 |  |
| Rabbit monoclonal NEK7 | HUABIO | Cat#ET7110-16 |  |
| Rabbit monoclonal GSDMD | HUABIO | Cat#ER1901-37 |  |
| Rabbit monoclonal ASS1 | HUABIO | Cat#HA601101 |  |
| Rabbit monoclonal JAK2（Phospho Y1007+Y1008) | HUABIO | Cat#ET1607-34 |  |
| Rabbit monoclonal JAK2 | HUABIO | Cat#ET1607-35 |  |
| Rabbit monoclonal STAT1 | HUABIO | Cat#M1407-1 |  |
| Rabbit monoclonal Phospho-STAT1(S727) | HUABIO | Cat#ET1611-20 |  |
| Rabbit monoclonal CD36 | HUABIO | Cat#ET1607-38 |  |
| Rabbit monoclonal KEAP1 | ZENBIO | Cat#R26935 |  |
| Rabbit monoclonal GAPDH | HUABIO | Cat#ET1601-4 |  |
| Rabbit polyclonal TUBULIN | HUABIO | Cat#ET1601-4 |  |
| Rabbit polyclonal VINCULIN | HUABIO | Cat#ET1705-94 |  |
| Mouse monoclonal ASC | Santa Cruz Biotechnology | Santa Cruz Biotechnology |  |
| Rabbit monoclonal MPO | Abcam | Cat#ab208670 |  |
| Rabbit monoclonal NRF2 | HUABIO | Cat#R1312-8 |  |
| Rabbit monoclonal Phospho-NRF2(S40) | HUABIO | Cat#ET1608-28 |  |
| Rabbit polyclonal Ly6G | HUABIO | Cat#ET0809-11 |  |
| Rabbit monoclonal CD68 | Boster | Cat#BA3638 |  |
| Rabbit monoclonal Pacific Blue CD11b | ThermoFisher | Cat#RM2828 |  |
| Rabbit monoclonal APC-Gr1 | Biolegend | Cat#108411 |  |
| Rabbit monoclonal PE-F4/80 | BD BioScience | Cat# 565410 |  |
| Rabbit monoclonal FITC-CD45 | BD BioScience | Cat# 553080 |  |

**Table 2 The targeted siRNA sequence for mice Keap1**

| Gene | Sense (5’-3’) | Antisense (5’-3’) |
| --- | --- | --- |
| Negative control | UUCUCCGAACGUGUCACGUTT | ACGUGACACGUUCGGAGAATT |
| Mice Keap1 | GCCCAAUUCAUGGCUCACAAATT | UUUGUGAGCCAUGAAUUGGGCTT |

**Table 3 Thesequences of primer for PCR amplification**

| Gene | Forward primer (5’-3’) | Reverse primer (5’-3’) |
| --- | --- | --- |
| Mice IL-1β | ACGGACCCCAAAAGATGAAG | TTCTCCACAGCCACAATGAG |
| Mice IL-6 | CAAAGCCAGAGTCCTTCAGAG | GTCCTTAGCCACTCCTTCTG |
| Mice TNFα | CTTCTGTCTACTGAACTTCGGG | CAGGCTTGTCACTCGAATTTTG |
| Mice NOS2 | GCAAACATCACATTCAGATCCC | TCAGCCTCATGGTAAACACG |
| Mice COX2 | CTCACGAAGGAACTCAGCAC | GGATTGGAACAGCAAGGATTTG |
| Mice SQSTM1 | CCTATACCCACATCTCCCACC | TGTCGTAATTCTTGGTCTGTAGG |
| Mice Prdx5 | AAGCAGGTTGGGAGTGTG | TTCCCCTTCAAATACCTCCAC |
| Mice GAPDH | CTTTGTCAAGCTCATTTCCTGG | TCTTGCTCAGTGTCCTTGC |

**Table 4 The information about the genes encoded by the DEPs**

| **Gene Name** | **ID** | **Log2Fold change** | | **P-value** |
| --- | --- | --- | --- | --- |
| Tmprss13 | Q5U405 | | 1.719324873 | 2.75E-02 |
| Pcyox1l | Q8C7K6 | | 1.406761488 | 2.69E-02 |
| Derl1 | Q99J56 | | 1.399761647 | 1.50E-02 |
| Spp1 | P10923 | | 1.362219545 | 1.85E-02 |
| Hic2 | Q9JLZ6 | | 1.307199562 | 4.38E-02 |
| Ssr2 | Q9CPW5 | | 1.304459795 | 3.36E-02 |
| Pyurf | Q9D1C3 | | 1.298884254 | 4.21E-03 |
| Adam8 | Q05910 | | 1.294796984 | 1.07E-02 |
| Uqcr11 | Q9CPX8 | | 1.289616185 | 6.45E-03 |
| Fam241a | Q9CZL2 | | 1.280330499 | 2.59E-02 |
| Ucp2 | P70406 | | 1.279453385 | 1.78E-02 |
| Arf3 | P61205 | | 1.275948614 | 1.09E-02 |
| Mpv17 | P19258 | | 1.263207847 | 5.36E-03 |
| Tapt1 | Q4VBD2 | | 1.256364532 | 1.07E-02 |
| Dhrs7 | Q9CXR1 | | 1.23370534 | 1.64E-03 |
| Adipor1 | Q91VH1 | | 1.232163116 | 1.31E-03 |
| Tmem41b | Q8K1A5 | | 1.22907494 | 2.68E-02 |
| Micu2 | Q8CD10 | | 1.225226124 | 3.30E-02 |
| H2-Q10 | P01898 | | 1.224576808 | 9.22E-03 |
| Exosc5 | Q9CRA8 | | 1.221607063 | 3.43E-04 |
| Fgfr1op | Q66JX5 | | 1.216449946 | 8.28E-03 |
| Fam210a | Q8BGY7 | | 1.209959978 | 3.91E-02 |
| Slc15a3 | Q8BPX9 | | 1.206594346 | 6.83E-03 |
| Prdx5 | P99029 | | 1.206281883 | 1.63E-02 |
| Dab2 | P98078 | | 1.20458186 | 6.99E-03 |
| Uvssa | Q9D479 | | 0.830882344 | 1.70E-02 |
| Ube2d3 | P61079 | | 0.826358829 | 3.31E-03 |
| Pfkfb2 | P70265 | | 0.82462034 | 4.31E-02 |
| Rgmb | Q7TQ33 | | 0.814278313 | 3.61E-02 |
| Irf7 | P70434 | | 0.81271367 | 3.17E-03 |
| Ube2d2 | P62838 | | 0.805035117 | 3.32E-02 |
| Bub1 | O08901 | | 0.804116087 | 2.94E-02 |
| Sptb | P15508 | | 0.788989313 | 2.68E-02 |
| Hist2h2ac | Q64523 | | 0.777693558 | 2.67E-02 |
| Idi2 | Q8BFZ6 | | 0.767180869 | 4.51E-02 |
| Tecpr1 | Q80VP0 | | 0.754909515 | 3.08E-02 |
| Hist2h2aa1 | Q6GSS7 | | 0.750950191 | 1.17E-02 |
| Ccdc117 | Q6PB51 | | 0.741886414 | 8.38E-03 |
| Kdm6b | Q5NCY0 | | 0.741597347 | 1.88E-02 |
| Lclat1 | Q3UN02 | | 0.740456906 | 4.74E-02 |
| Hbb-b1 | P02088 | | 0.734130629 | 3.41E-02 |
| Adam23 | Q9R1V7 | | 0.726070961 | 2.50E-02 |
| Mfsd14a | P70187 | | 0.699078542 | 3.43E-02 |
| Slc13a5 | Q67BT3 | | 0.661197098 | 3.58E-02 |
| Hist1h2af | Q8CGP5 | | 0.600187696 | 2.77E-02 |
| Gabarap | Q9DCD6 | | 0.5463331 | 3.12E-02 |
| Tubb4a | Q9D6F9 | | 0.528679414 | 3.26E-02 |
| Ifi205a | Q8CGE8 | | 0.50908055 | 1.14E-03 |

**Supplementary figure and figure legends**

**
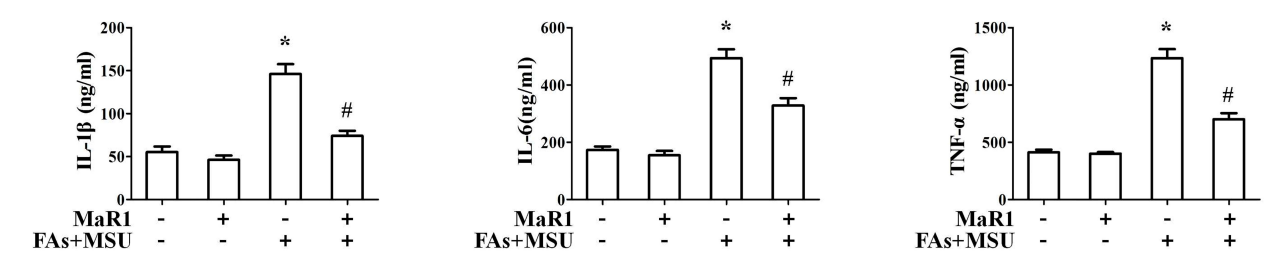
**

**Sup Fig. 1** MaR1 inhibits the secretion of IL-1β, IL-6, TNF-α. ELISA assay was used to detect IL-1β, IL-6, TNF-α, TGF-β and IL-10 levels in cell culture supernatants. * compared with no FAs + MSU crystals treatment, # compared with FAs + MSUc treatment. * and # means P<0.05.


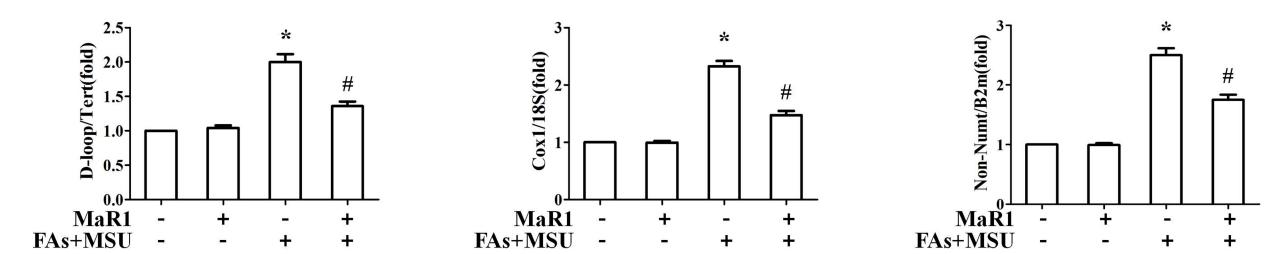


**Sup Fig. 2**Relative total mtDNA amounts in BMDMs treated with or without MaR1 for 1 h, followed by FAs + MSUc for 12 h. Shown are the ratio of D-loop mtDNA to Tert nuclear (n) DNA, Cox1 mtDNA to 18S nDNA or mtDNA that is not inserted into nuclear DNA (non-NUMT) to B_2_m nDNA.* compared with no FAs + MSUc treatment, # compared with FAs + MSUc treatment. * and # means P<0.05.


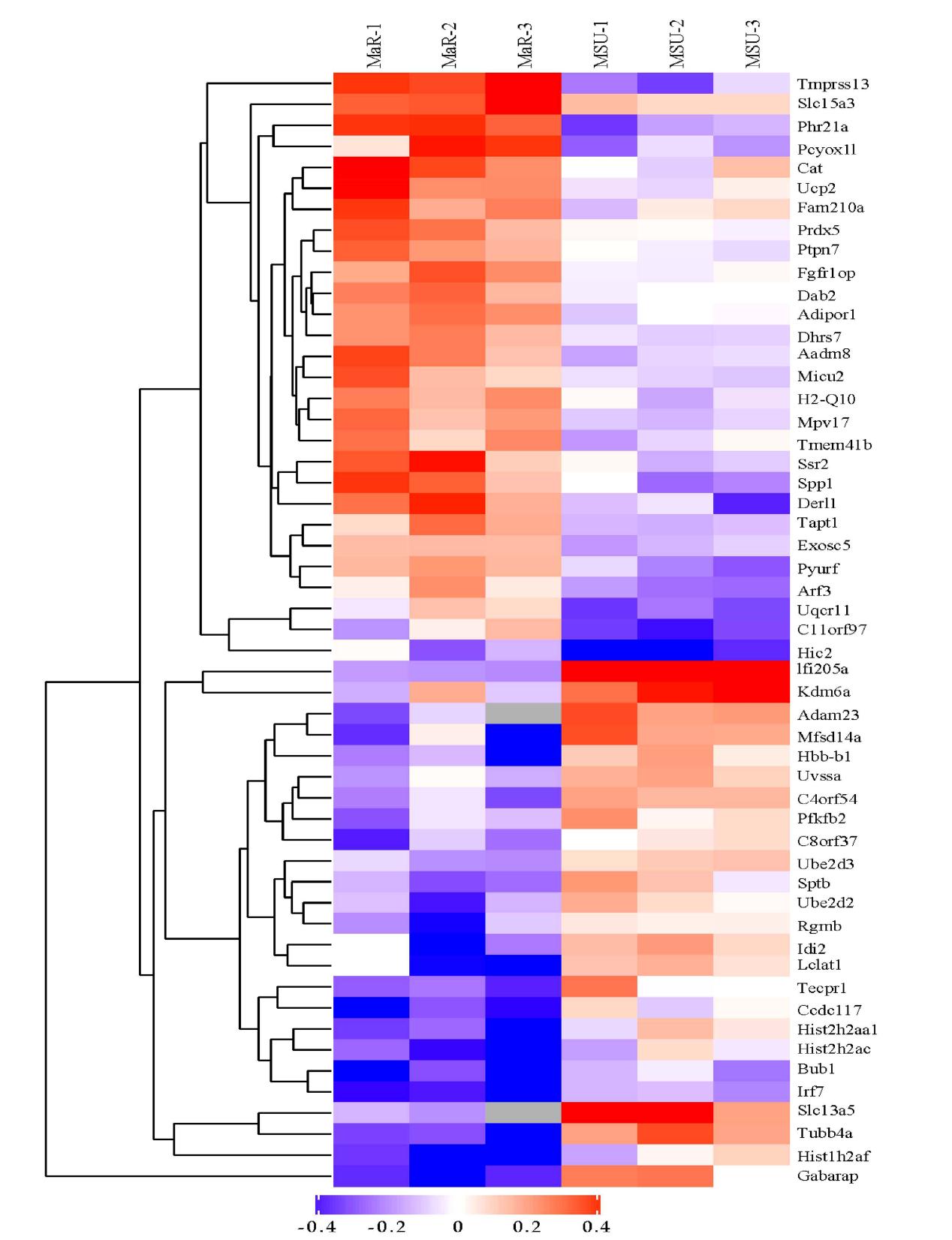


**Sup Fig. 3**Expression analysis of differentially expressed proteins (DEPs) in BMDMs treated with or without MaR1 for 1 h, followed by FAs + MSUc for 12 h. Heat-map was used to show the DEGs. The colors ranging from red to blue indicate the protein levels from high to low. MSU-1, MSU-2 and MSU-3 represent FAs + MSUc treatment groups, MaR-1, MaR-2 and MaR-3 represent MaR1 and FAs + MSUc treatment groups. N=3 each group.


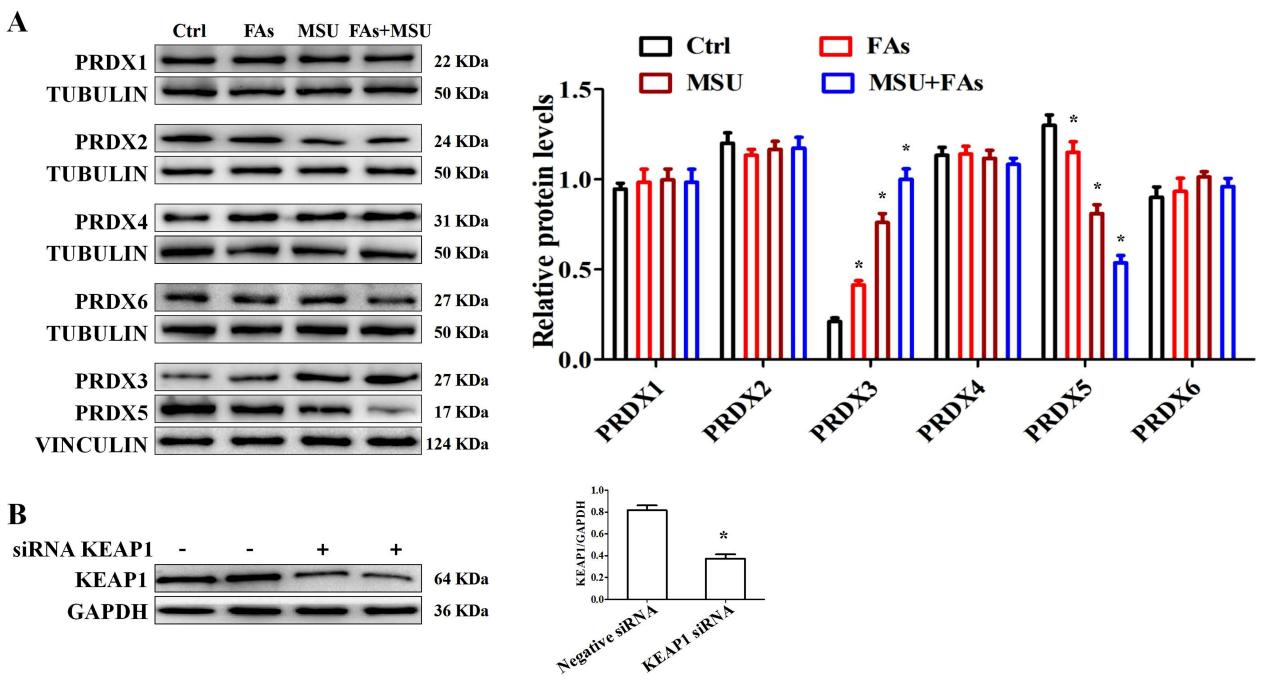


**Sup Fig. 4 Effect of FAs, MSU crystals, FAs + MSUc stimulation on PRDX1-6 protein levels in BMDMs and targeting Keap1 siRNA to inhibit KEAP1 protein expression. (A)**BMDMs were treated with FAs, MSUc or FAs + MSUc for 12 h. Western blot detection of PRDX1-6 protein levels. * compared with Vehicle (BSA) treatment. (**B)**Western blot detection of KEAP1 protein level. *compared to BMDMs transfected with negative control siRNAs. * means P<0.05.


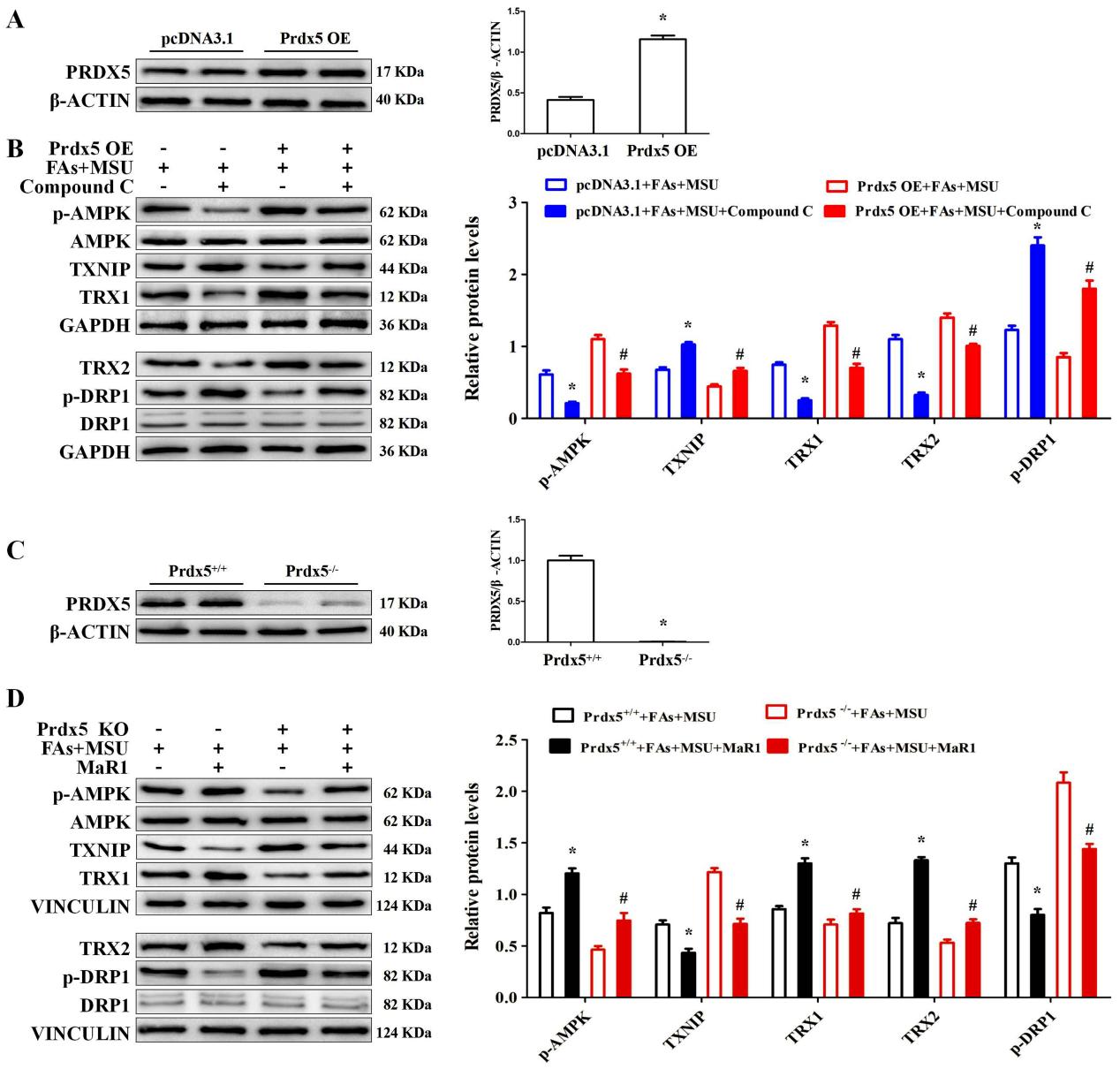


**Sup Fig. 5 PRDX5 protein expression and Compound C reversed the effect of Prdx5 overexpression on AMPK activation and downstream target proteins. (A)**BMDMs were transfected with pcDNA3.1 or Prdx5 ORF was cloned into pcDNA3.1 plasmid.Western blot for PRDX5 protein level. *compared with transfection pcDNA3.1 plasmid. (**B)**Western blot for p-AMPK, TXNIP, TRX1,TRX2 and p-DRP1protein levels.*compared with pcDNA3.1 + FAs + MSU crystals. # compared with Prdx5 OE + FAs + MSUc. (**C)**Detection of PRDX5 protein level in BMDMs derived from Prdx5^+/+^ and Prdx5^-/-^ mice.*compared with BMDMs derived from Prdx5^+/+^. * and # means P<0.05. (**D)**BMDMs from Prdx5^+/+^ and Prdx5^-/-^ mice were treated with or without MaR1, then stimulated with FAs + MSUc. Western blot for p-AMPK, TXNIP, TRX1, TRX2 and p-DRP1 protein levels. *compared with Prdx5^+/+^ + FAs + MSUc. # compared with Prdx5^+/+^ + FAs + MSU crystals + MaR1. * and # means P<0.05.


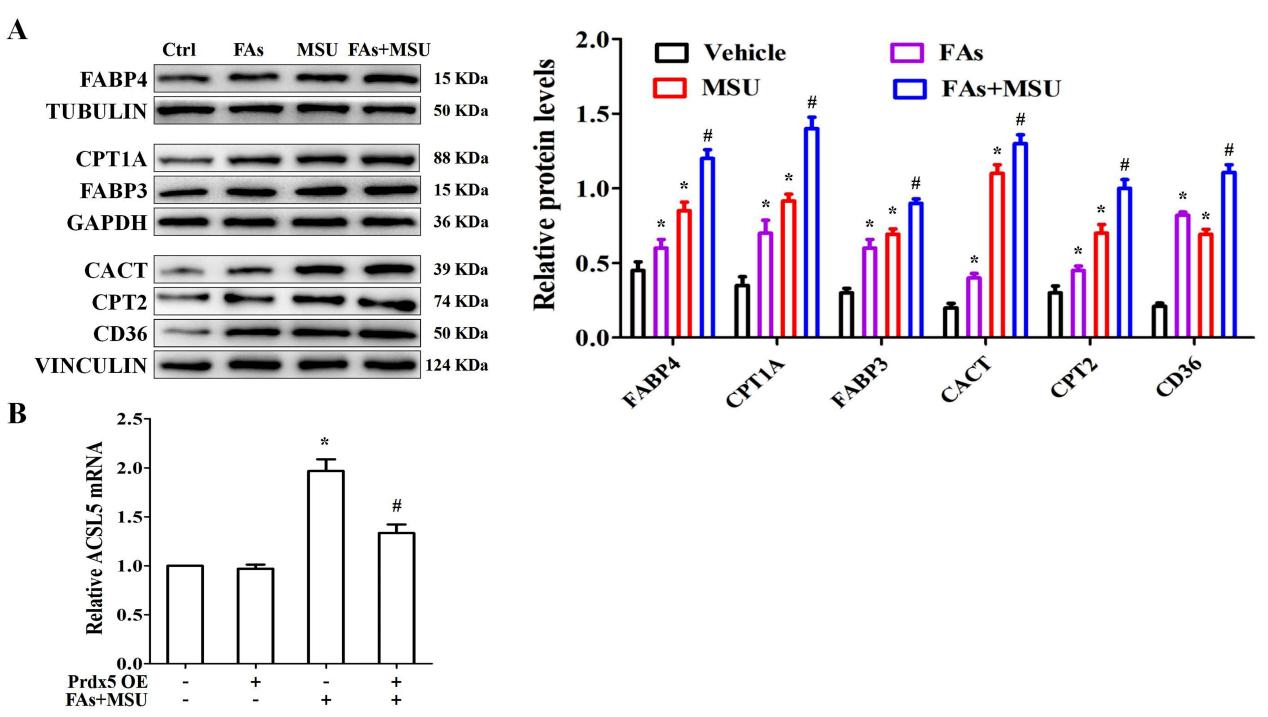
**Sup Fig. 6 Effects of FAs, MSUc and FAs + MSU crystals stimulation on the expression of FAO related genes. (A)** BMDMs were treated with FAs, MSUc or FAs + MSUc for 12 h. Western blot detection of FABP4, CPT1A, FABP3, CACT, CPT2 and CD36 protein levels. * compared with no FAs, MSUc and FAs + MSUc treatment. * means P<0.05. (**B)**Quantitative PCR to detect the mRNA level of ACSL5. * compared with no FAs + MSU crystals treatment, # compared with FAs + MSUc treatment. * and # means P<0.05.


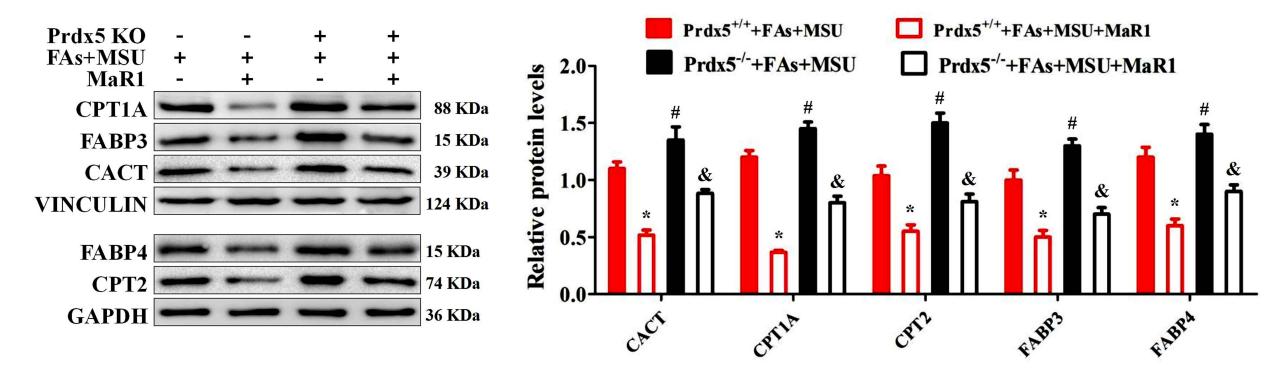


**Sup Fig. 7 MaR1 affects AMPK activity mainly through upregulation Prdx5 expression.**BMDMs from Prdx5^+/+^ and Prdx5^-/-^ mice were treated with or without MaR1, then stimulated with FAs + MSUc. Western blot for CPT1A, FABP3,CACT, FABP4 and CPT2 protein levels. *compared with Prdx5^+/+^ + FAs + MSUc. # compared with Prdx5^+/+^ + FAs + MSUc + MaR1. * and # means P<0.05.


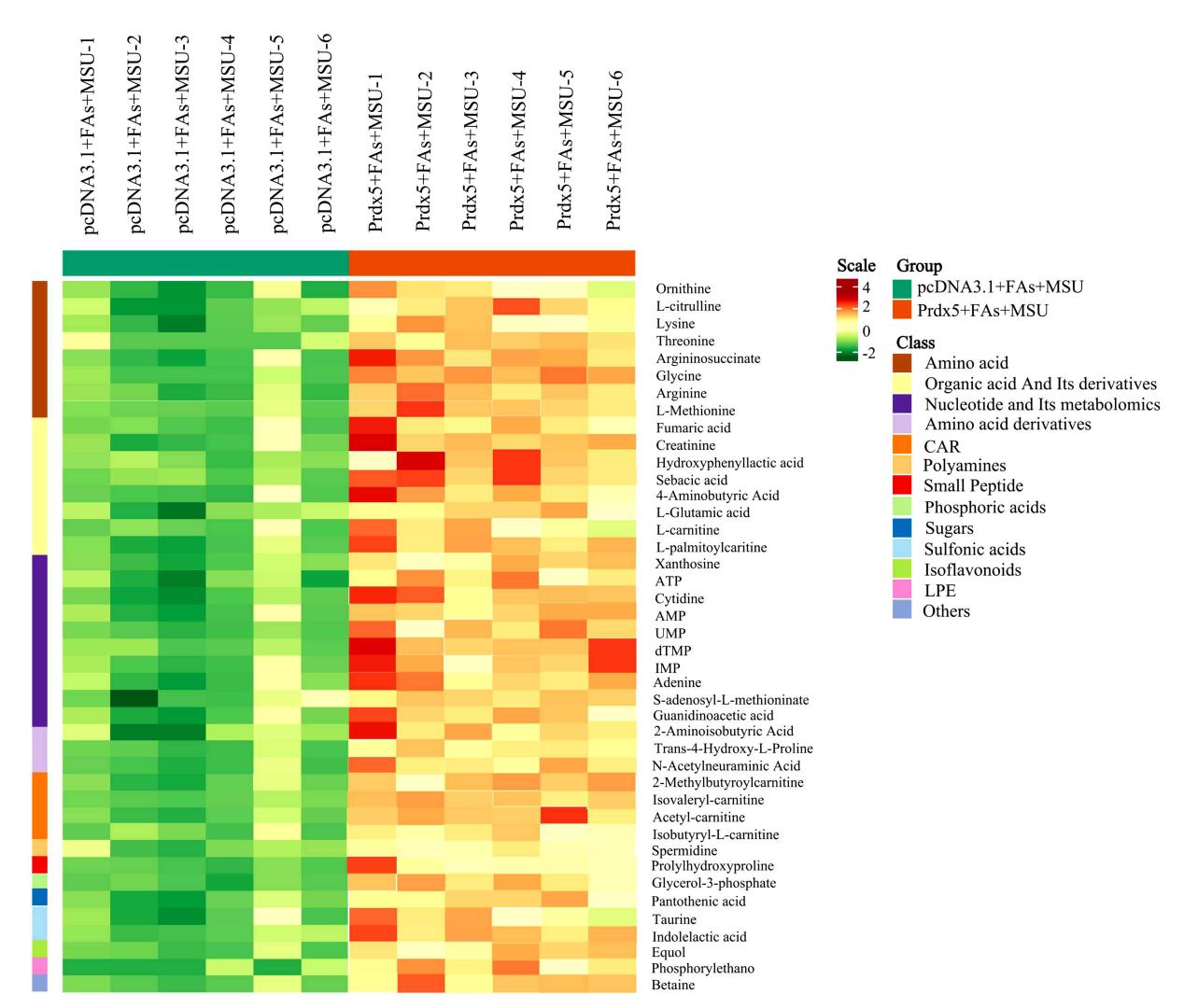


**Sup Fig. 8 The heat-map showed the effects of FAs + MSUc on metabolites in BMDMs.**

**
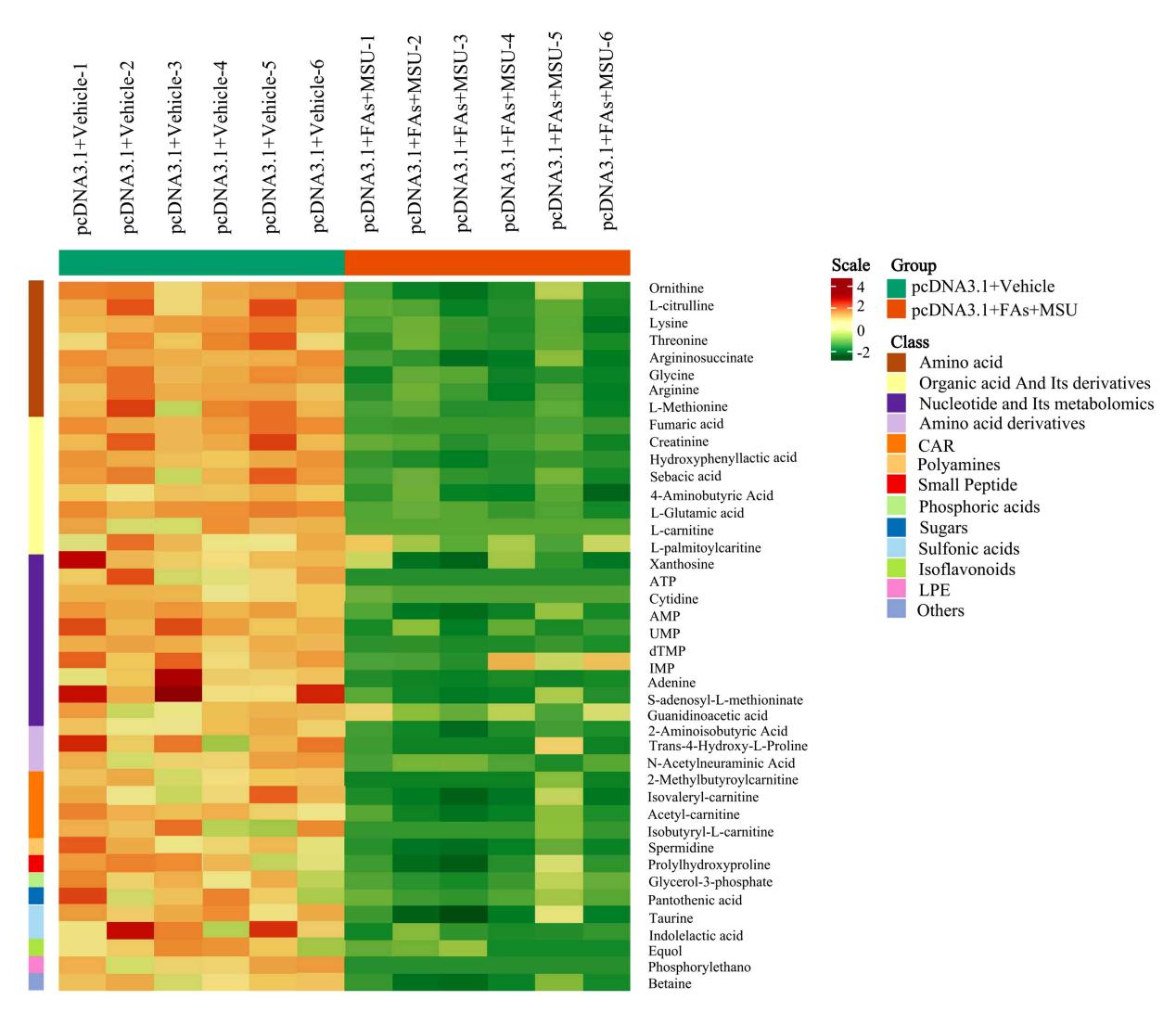
**

**Sup Fig. 9 The heatmap listed the effects of Prdx5 overexpression on metabolites in BMDMs treated with FAs + MSUc.**

**
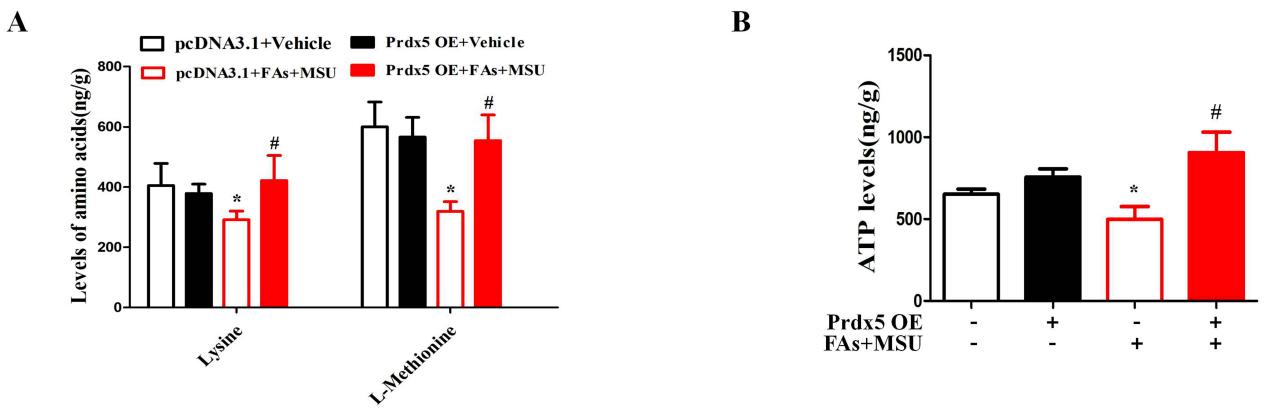
**

**Sup Fig. 10 Prdx5 overexpression increases Lysine, L-Methionine and ATP production. (A)** The levels of Lysine and L-Methionine. **(B)** The levels of ATP. *compared with Vehicle (BSA) treatment, # compared with pcDNA3.1 + FAs + MSUc.


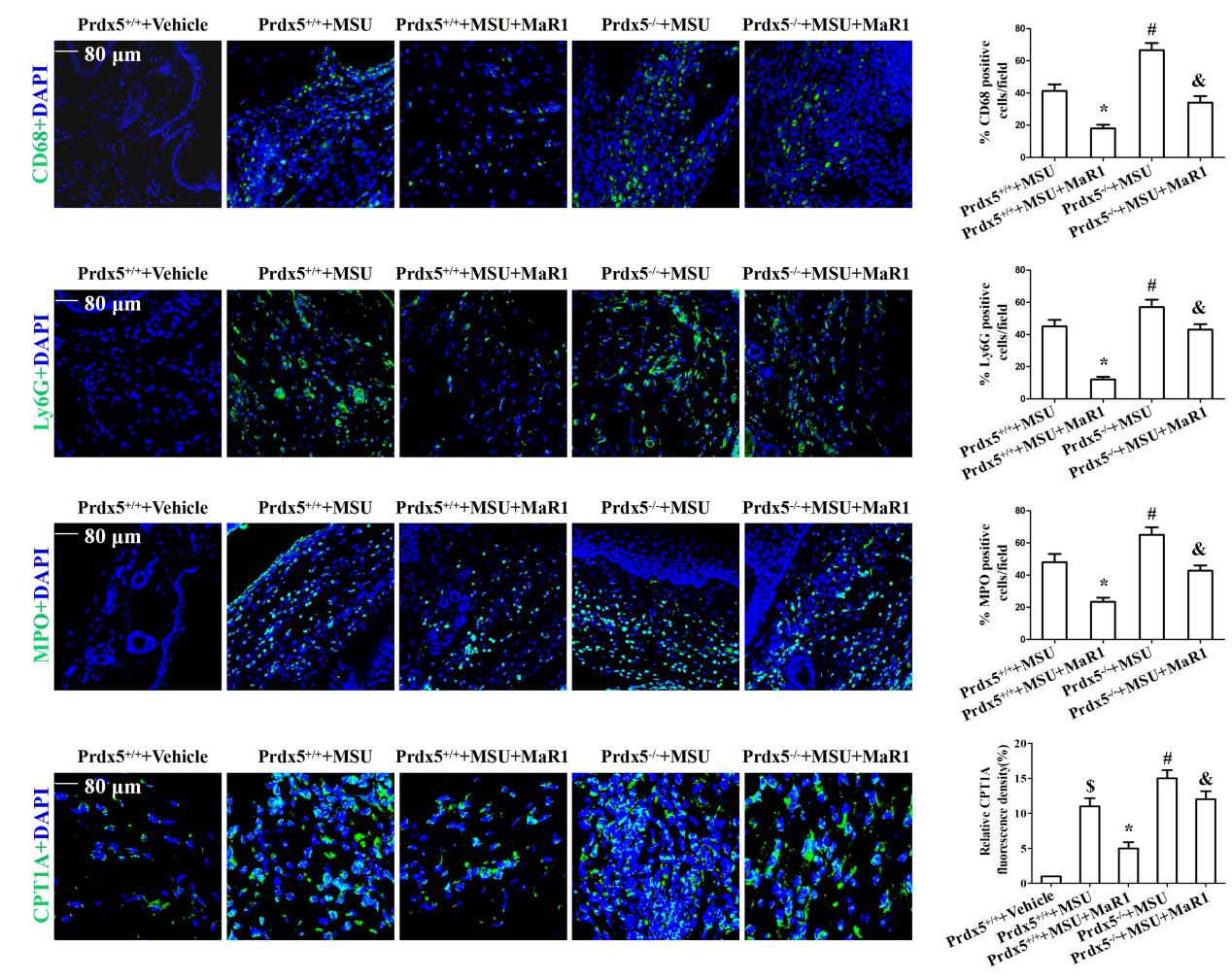


**Sup Fig.11. MaR1 treatment inhibited the distribution of CD68, Ly6G, MPO and CPT1A proteins induced by MSU crystals in mice footpad tissue section.** Immunofluorescence detection of CD68, Ly6G, MPO and CPT1A protein distribution. *compared with Prdx5^+/+^ + MSU, # compared with Prdx5^+/+^ + MSU, & compared with Prdx5^+/+^ + MSU, $ compared with Prdx5^+/+^ + MSU + MaR1. *, # and & means P<0.05.

**Original Western blot bands**


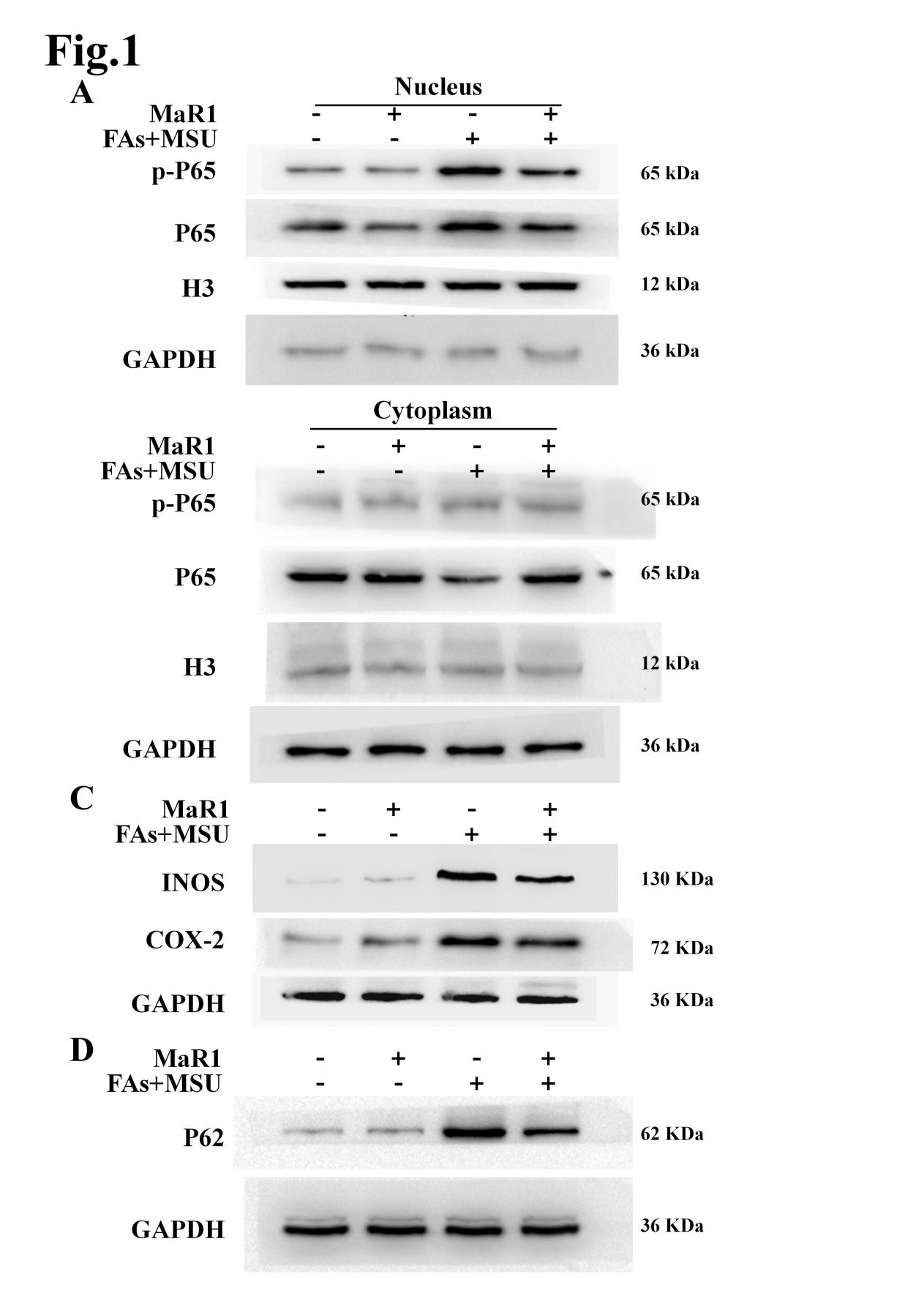


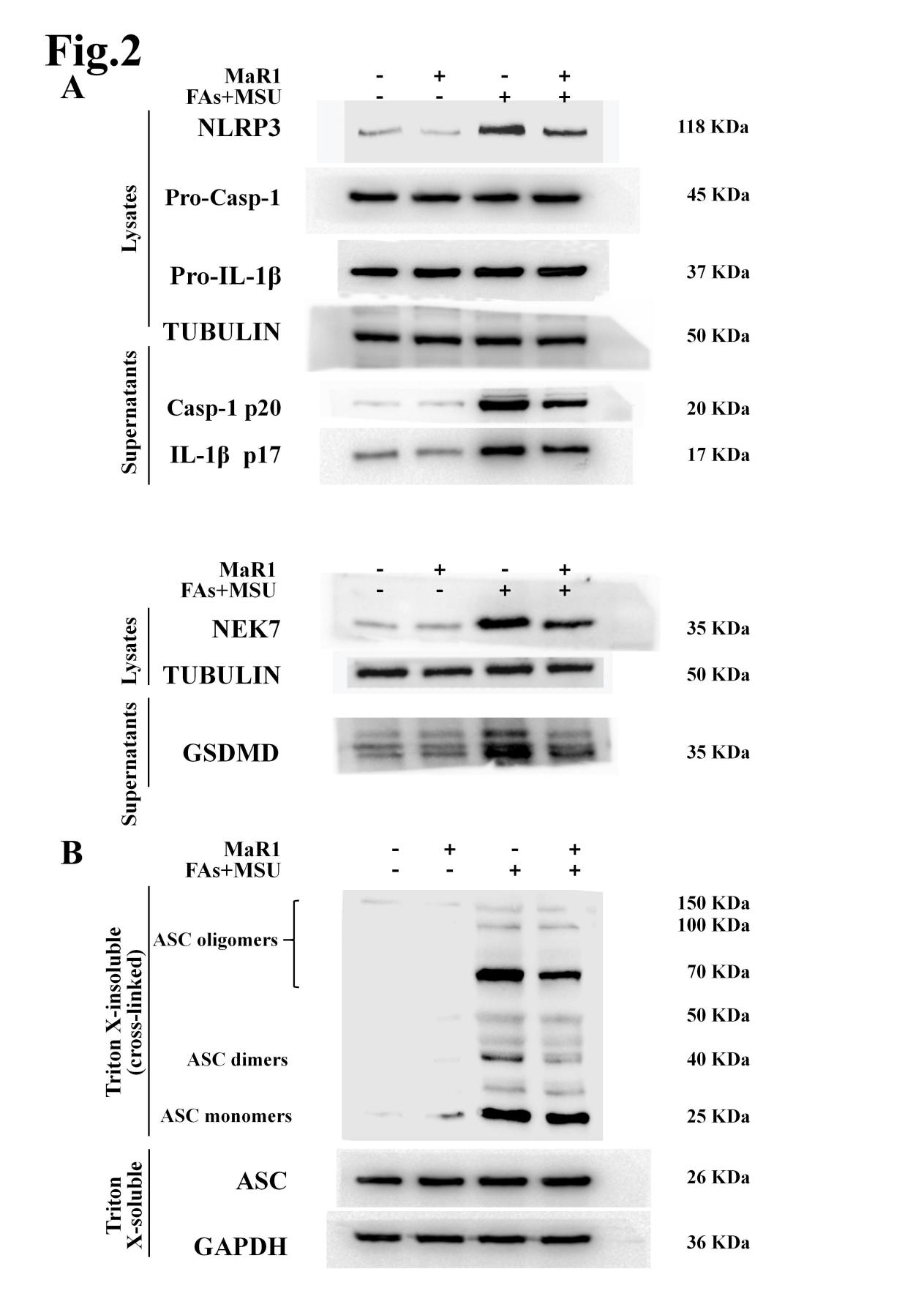


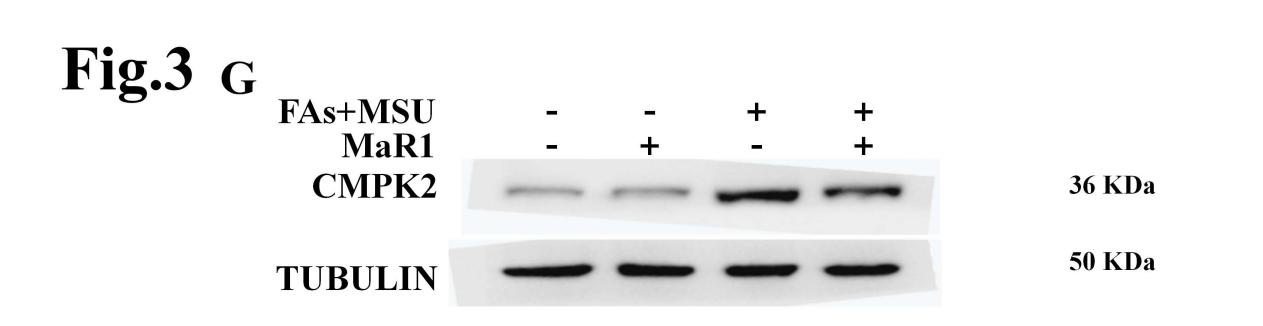


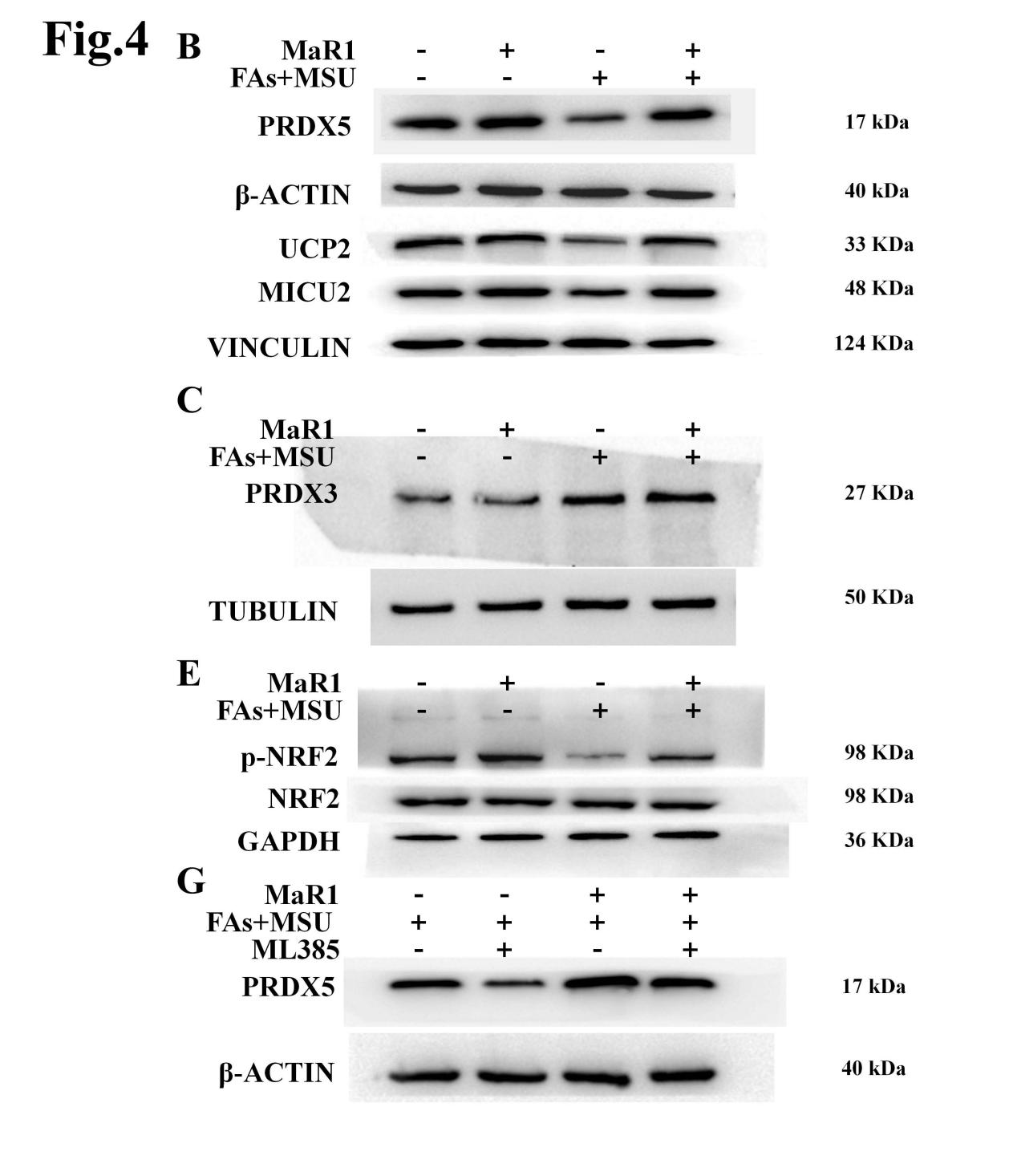


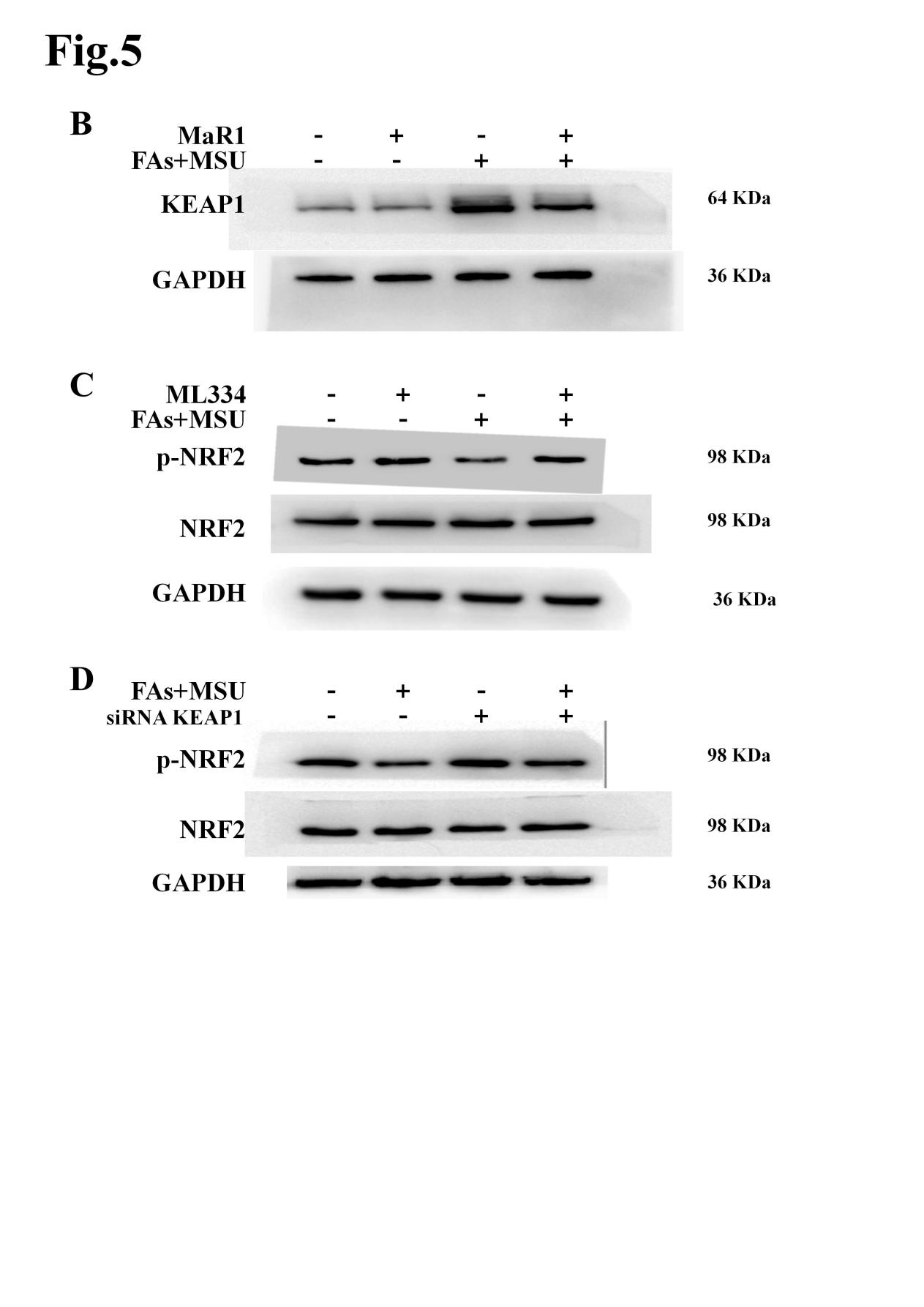


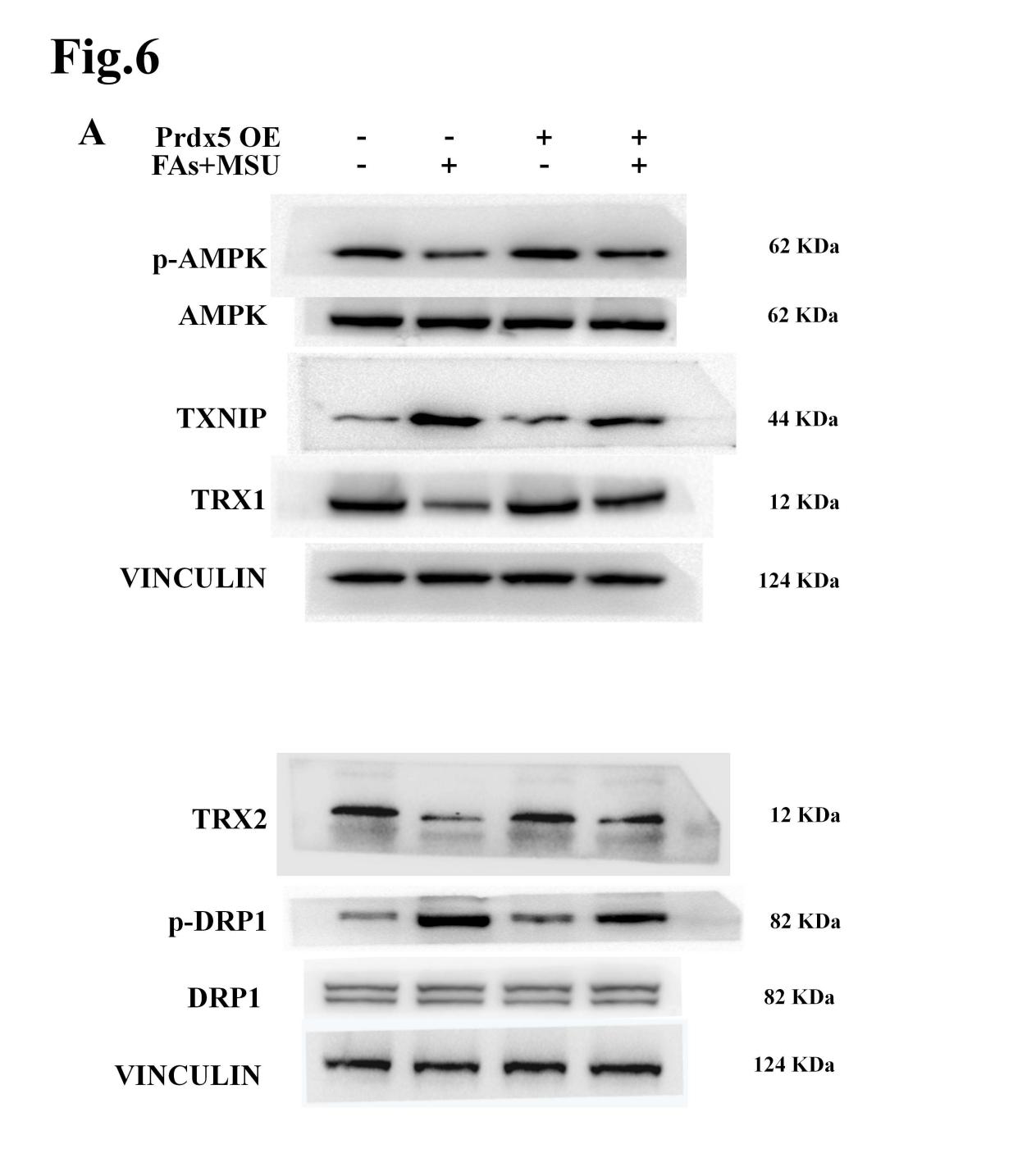


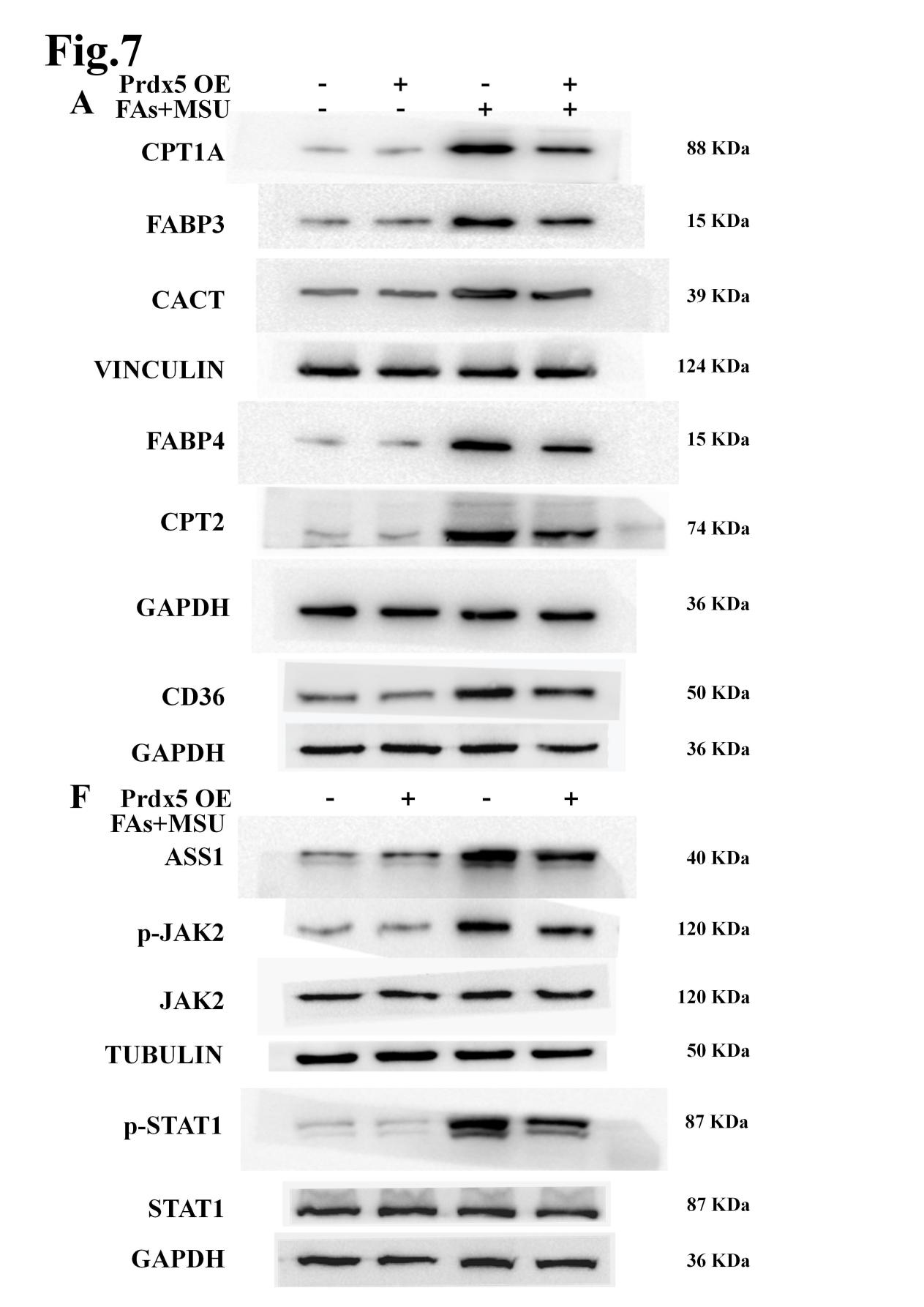


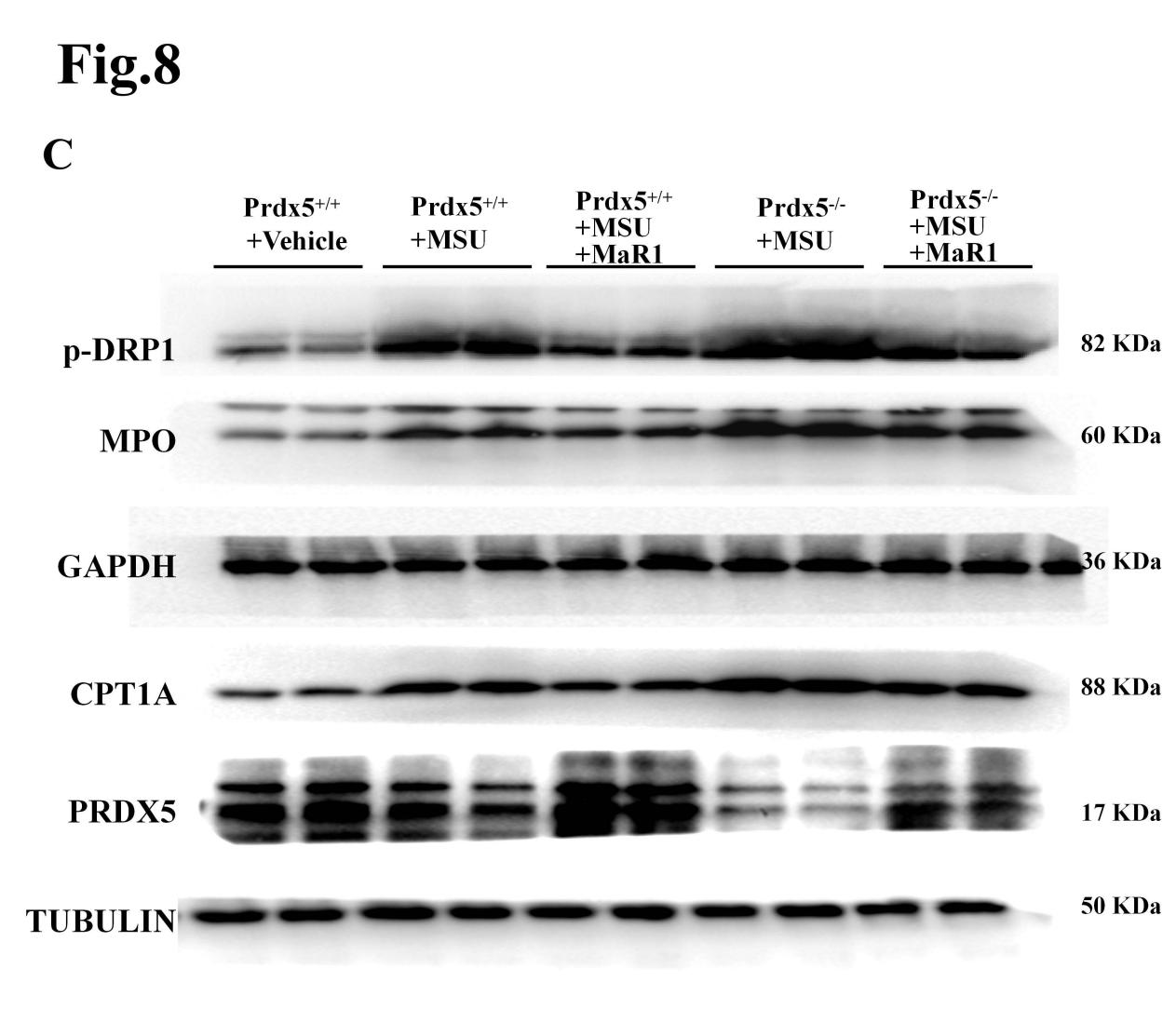


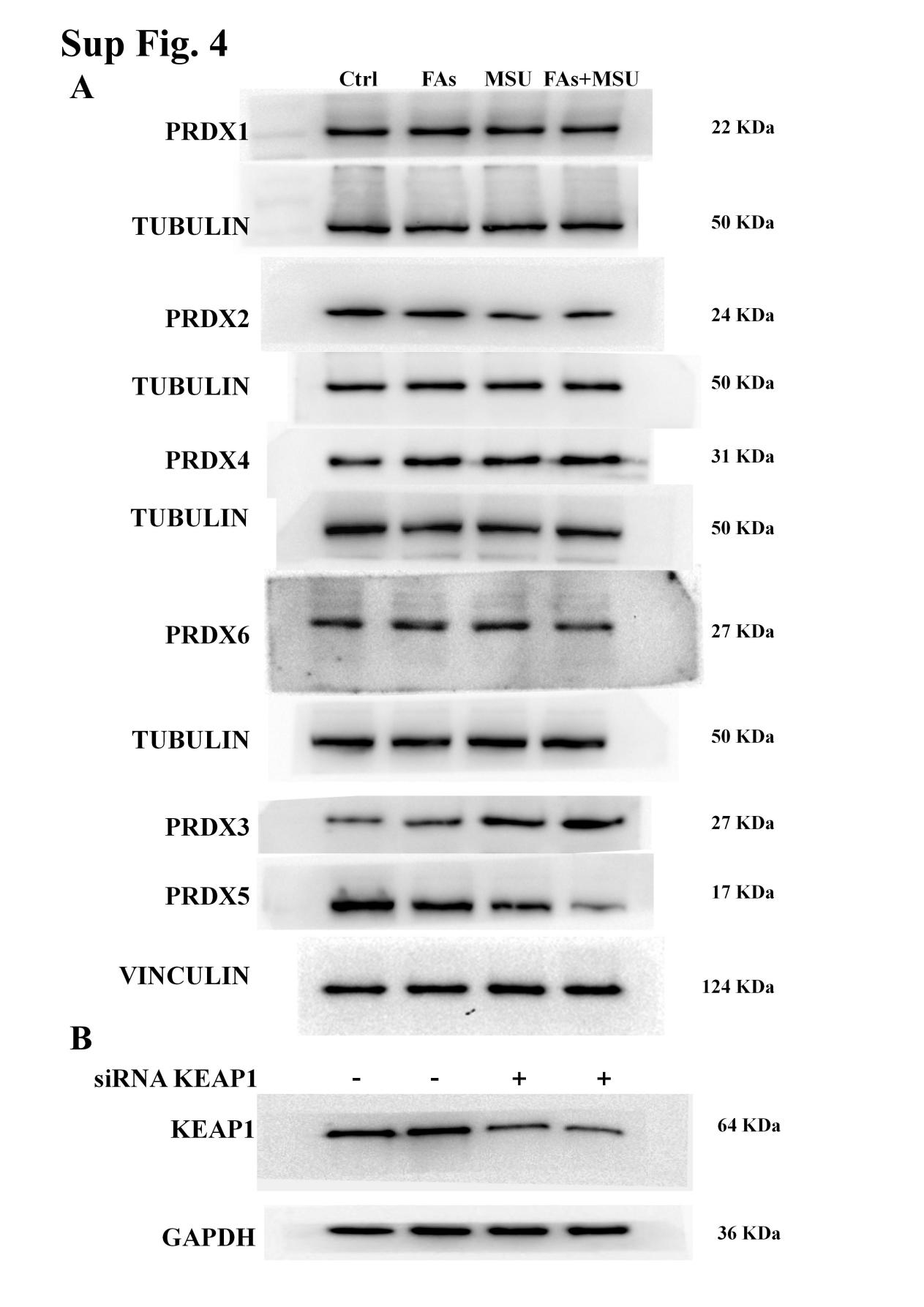


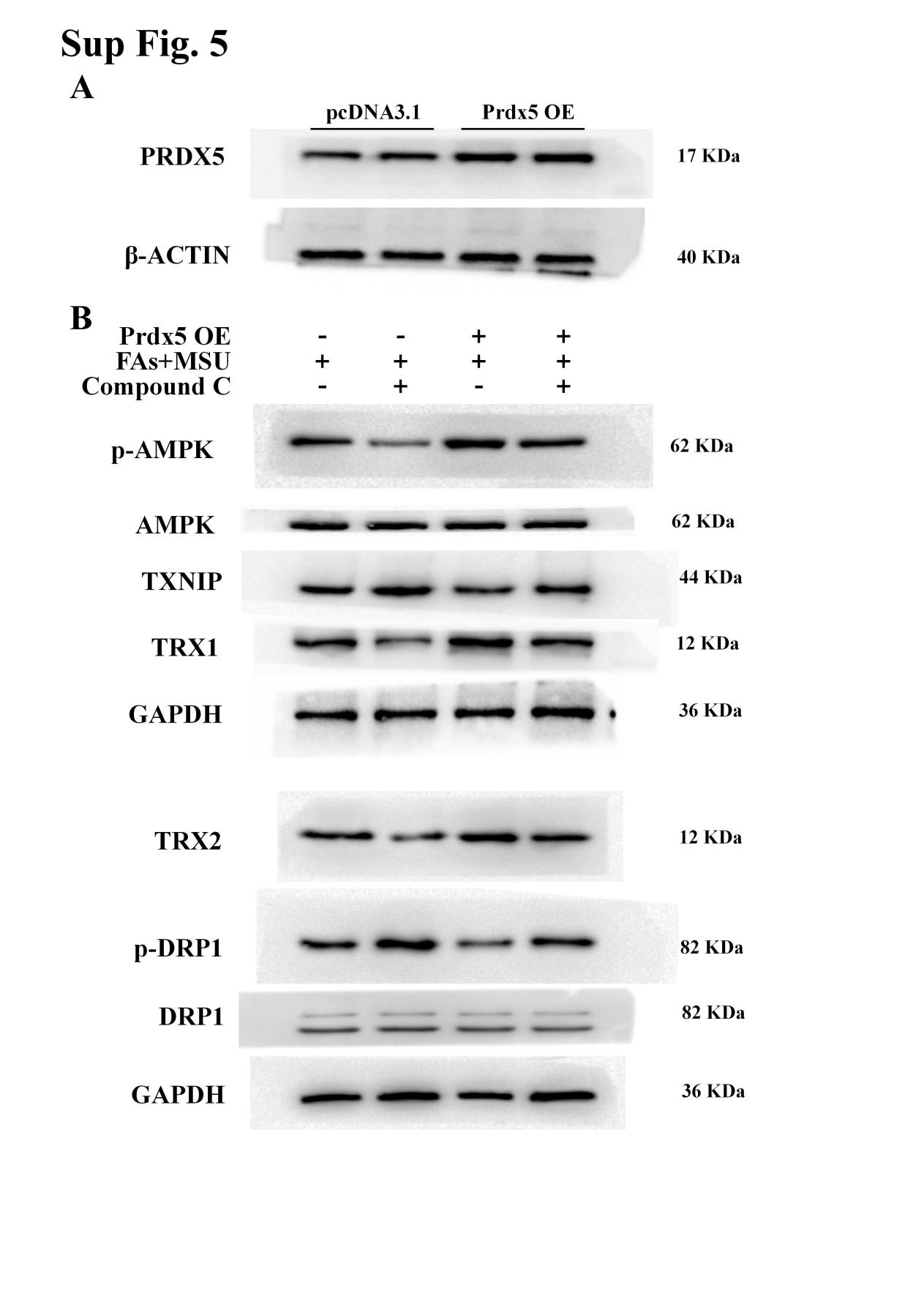


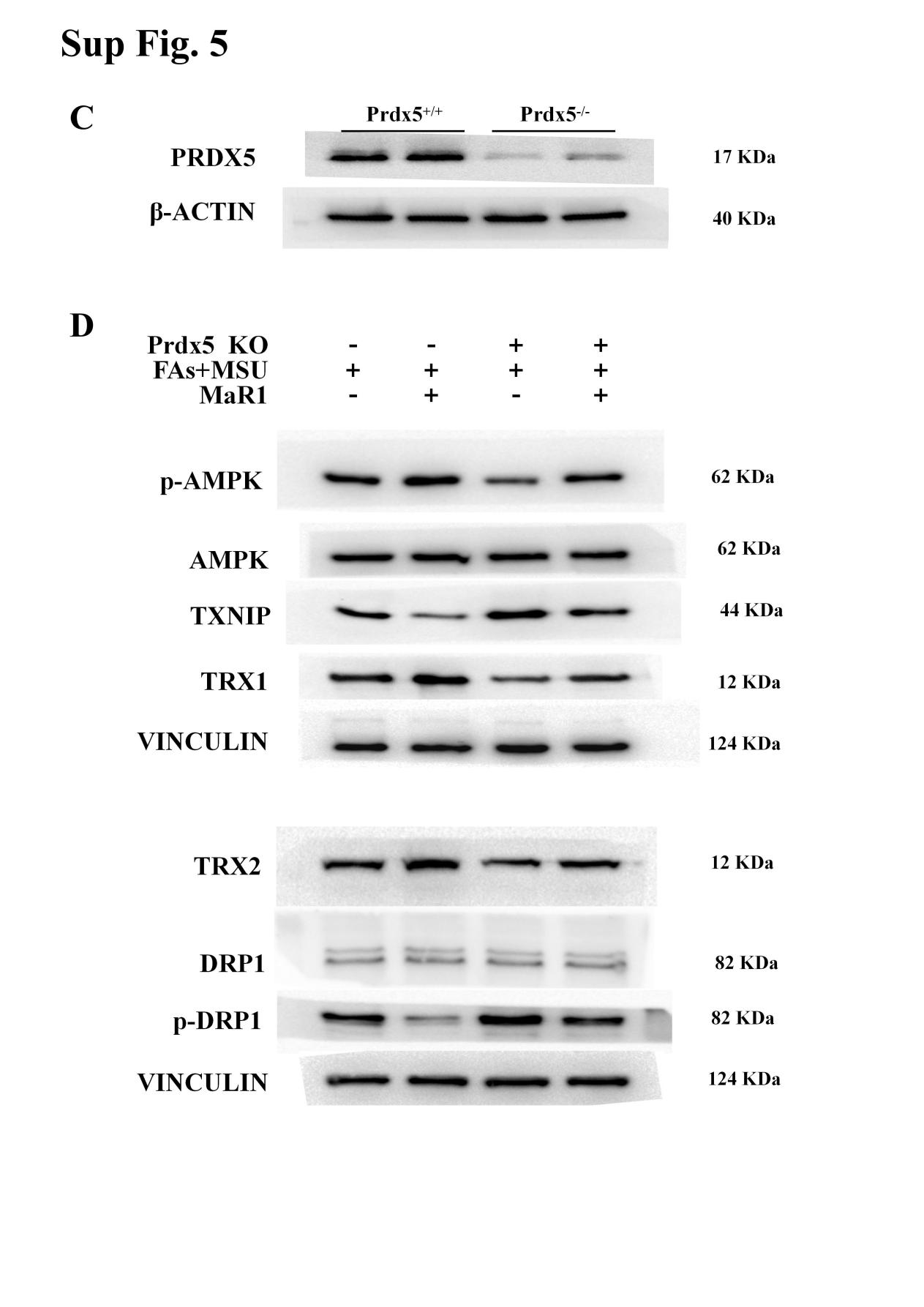


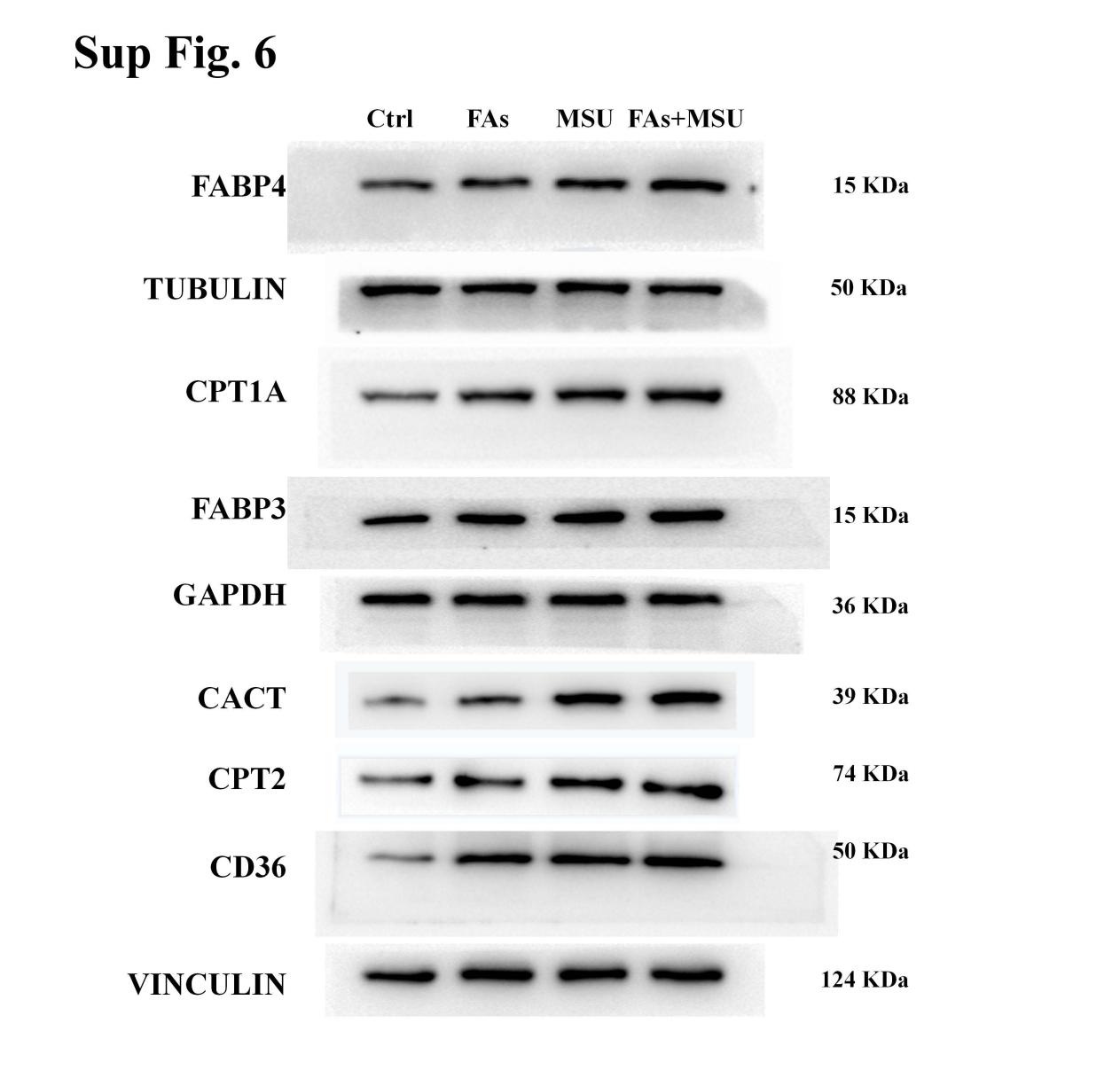


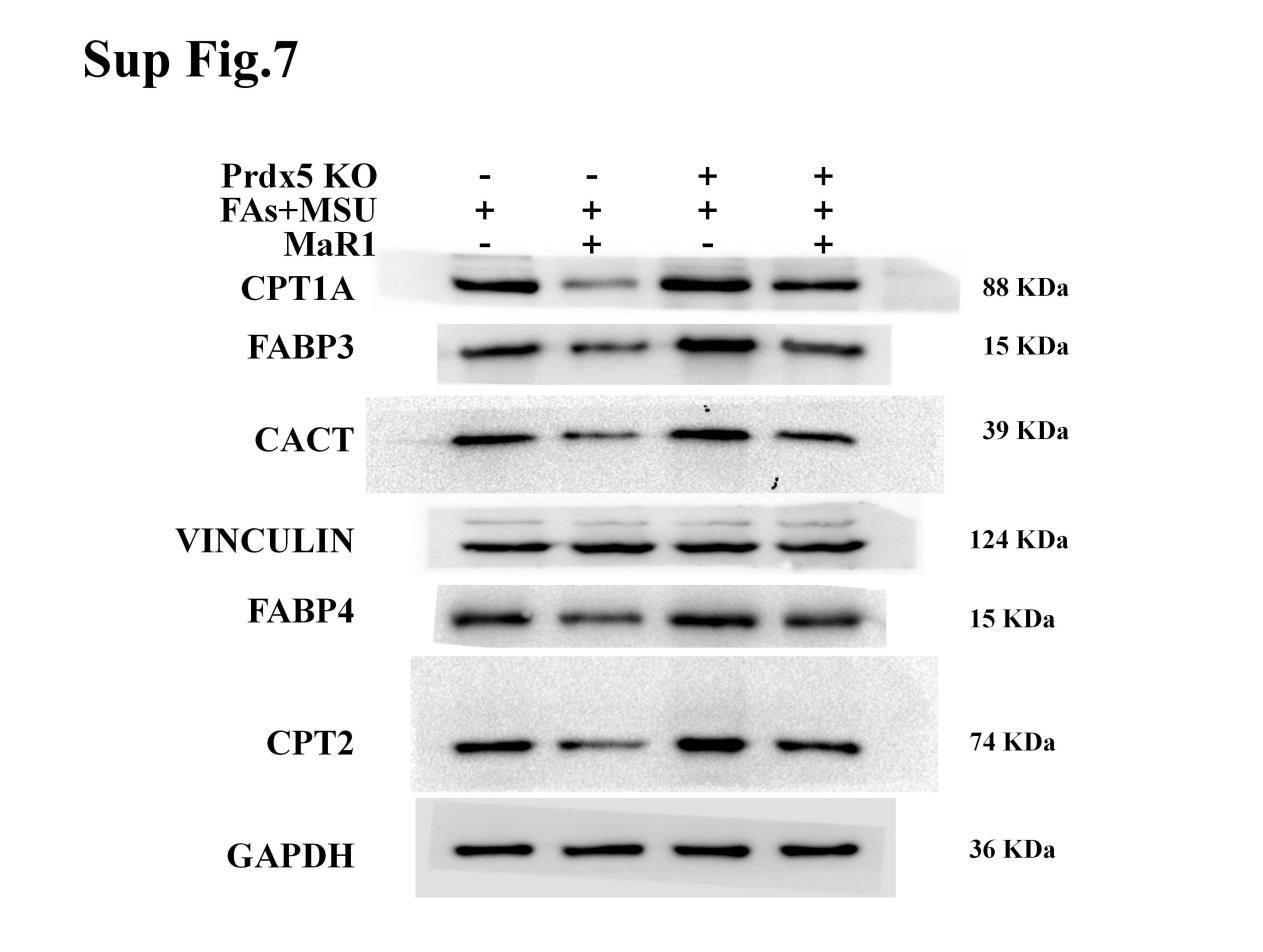

Supplement: Supplementary file 1 — Additional file 1: Table S1. The antibodies were used in Western blotting immunofluorescence assays.Table S2. The targeted siRNA sequence for mice Keap1. Table S3. The sequences of primer for PCR amplification.Table S4. The information about the genes encoded by the DEPs. Fig. S1. MaR1 inhibits the secretion of IL-1β, IL-6, TNF-α. ELISA assay was used to detect IL-1β, IL-6, TNF-α, TGF-β and IL-10 levels in cell culture supernatants. * compared with no FAs + MSU crystals treatment, # compared with FAs + MSUc treatment. * and # means P<0.05. Fig. S2. Relative total mtDNA amounts in BMDMs treated with or without MaR1 for 1 h, followed by FAs + MSUc for 12 h. Shown are the ratio of D-loop mtDNA to Tert nuclear (n) DNA, Cox1 mtDNA to 18S nDNA or mtDNA that is not inserted into nuclear DNA (non-NUMT) to B2m nDNA.* compared with no FAs + MSUc treatment, # compared with FAs + MSUc treatment. * and # means P<0.05. Fig. S3. Expression analysis of differentially expressed proteins (DEPs) in BMDMs treated with or without MaR1 for 1 h, followed by FAs + MSUc for 12 h. Heat-map was used to show the DEGs. The colors ranging from red to blue indicate the protein levels from high to low. MSU-1, MSU-2 and MSU-3 represent FAs + MSUc treatment groups, MaR-1, MaR-2 and MaR-3 represent MaR1 and FAs + MSUc treatment groups. N=3 each group. Fig. S4. Effect of FAs, MSU crystals, FAs + MSUc stimulation on PRDX1-6 protein levels in BMDMs and targeting Keap1 siRNA to inhibit KEAP1 protein expression. (A)BMDMs were treated with FAs, MSUc or FAs + MSUc for 12 h. Western blot detection of PRDX1-6 protein levels. * compared with Vehicle (BSA) treatment. (B)Western blot detection of KEAP1 protein level. *compared to BMDMs transfected with negative control siRNAs. * means P<0.05. Fig. S5. PRDX5 protein expression and Compound C reversed the effect of Prdx5 overexpression on AMPK activation and downstream target proteins. (A)BMDMs were transfected with pcDNA3.1 or Prdx5 ORF was cloned into pcDNA3.1 p [file 10020_2023_756_MOESM1_ESM.docx]
